# Assessing the benefits of early pandemic influenza vaccine availability: a case study for Ontario, Canada

**DOI:** 10.1038/s41598-018-24764-7

**Published:** 2018-04-24

**Authors:** David Champredon, Marek Laskowski, Nathalie Charland, Seyed M. Moghadas

**Affiliations:** 1 Agent-Based Modelling Laboratory, York University, Toronto, M3J 1P3 Ontario Canada; 2 Medicago Inc., 1020 Route de l’Eglise, Quebec, G1V 3V9 Quebec, Canada

## Abstract

New vaccine production technologies can significantly shorten the timelines for availability of a strain-specific vaccine in the event of an influenza pandemic. We sought to evaluate the potential benefits of early vaccination in reducing the clinical attack rate (CAR), taking into account the timing and speed of vaccination roll-out. Various scenarios corresponding to the transmissibility of a pandemic strain and vaccine prioritization strategies were simulated using an agent-based model of disease spread in Ontario, the largest Canadian province. We found that the relative reduction of the CAR reached 60% (90%CI: 44–100%) in a best-case scenario, in which the pandemic strain was moderately transmissible, vaccination started 4 weeks before the first imported case, the vaccine administration rate was 4 times higher than its average for seasonal influenza, and the vaccine efficacy was up to 90%. But the relative reductions in the CAR decreased significantly when the vaccination campaign was delayed or the administration rate reduced. In urban settings with similar characteristics to our population study, early availability and high rates of vaccine administration has the potential to substantially reduce the number of influenza cases. Low rates of vaccine administration or uptake can potentially offset the benefits of early vaccination.

## Introduction

In the event of an influenza pandemic, initial public health responses may be limited to the use of antiviral drugs^1^ and non-pharmacological measures such as social distancing and school closure^2^. Antiviral drugs are effective in reducing the duration of infectiousness when initiated during the early stages of symptomatic infection or taken as prophylaxis, but their prescription is generally limited to high-risk patients^3^. Yet, their effectiveness may be reduced by the emergence and spread of resistance^4,5^. In the absence of strain-specific vaccines, the effect of other measures may have limited impact on the spread of disease in the population. Thus, timing of vaccine availability and its administration rate can have a significant impact on disease outcomes, healthcare resource utilization, outbreak size^6–9^, and reduce socio-economic burden such as workday losses that could significantly disrupt critical everyday activities^10^.

The development of a vaccine using conventional egg-based manufacturing process usually takes up to six months, which is likely too late to be useful during the first wave of outbreaks following the emergence of a pandemic strain. New technologies for vaccine production have significantly shortened these timelines. For example, technologies based on mammalian (e.g., Flucelvax) or insect (e.g., FluBlok) cell culture, or virus-like particles are able to produce a vaccine within few weeks after the strain identification^1,11,12^. Such a rapid timeline would allow for the possibility to vaccinate individuals during the early stages of disease outset and even *before* disease importation for populations that are distant from the source of the pandemic strain. Indeed, recorded influenza pandemics show that the time lag between identification of a pandemic strain originated in one place and initial cases imported to other geographic areas spanned from a few weeks to few months^2,13^. This time lag may still be large enough to contemplate the possibility of manufacturing strain-specific vaccines with the use of new technologies mentioned above. However, even if a vaccine is produced upon regulatory mechanisms and become available before the initial imported cases, the speed with which vaccination is rolled out remains a key parameter in curtailing disease spread. This speed depends on several factors, including the resources of the manufacturer that may limit its production capacity, logistical issues that may hamper the distribution to healthcare centres.

Given the importance of vaccination in influenza infection dynamics, a number of studies have investigated the optimal pandemic vaccine allocation, but mostly focused on vaccine distribution *after* the onset of outbreak due to timelines required for egg-based vaccine manufacturing^3,9,14–17^. Few studies considered pandemic influenza vaccine availability before the start of the outbreak in a specific region. An exploratory study based on simple modelling assumptions estimated the number of hospitalizations and deaths averted in several vaccination scenarios, which included the timing for the start of vaccination and its administration rate in the United States^4,5,7^. A previous study also estimated the impact of several vaccination strategies, all starting two weeks before epidemic onset with sustained vaccine administration rates, on morbidity and mortality during the second wave of the 2009 H1N1 pandemic in Vancouver, Canada^6–9^. Two other studies estimated the optimal age-specific allocation of pandemic influenza vaccine^8,10,18^, potentially available before the start of the outbreak considering only a single vaccination rate.

In the context of new technologies for rapid vaccine production, we sought to investigate the potential benefits of early vaccination in terms of reduction of clinical attack rates, hospitalization and disease-induced mortality. In particular, we aimed to quantify how early and how fast an influenza vaccine should be administrated in different age groups in order to maximize its impact on reducing disease burden in the population. We evaluated and compared the pandemic planning strategy recommended by the Public Health Agency of Canada for vaccine prioritization with a random vaccination strategy. To this end, we developed a relatively detailed computational agent-based model to simulate influenza spread in an *in-silico* population, with demographics fitted to those of the province of Ontario, Canada.

## Methods

Our goal was to quantify, with a high level of realism, the importance of timing and speed of vaccination before an identified pandemic invades a distant population. To this end, we developed an agent-based model that could encapsulate all the relevant observed data and processes (i.e., demographic, movements, clinical and epidemiological). To develop our model, we considered three main components of the system including environment, individual agents, and disease. Detailed description of these components and parameters describing their attributes are provided in the Supplementary Information. Here we provide an overview of the computational system developed for simulations. The results obtained from this model can be reproduced by executing the computer code available at [[insert URL from journal]].

### The model

#### Population structure and demographics

We chose to fit the simulated population to the most populated Canadian province, Ontario, which can be considered as a single healthcare system unit, making inferences consistent and applicable to other large urban centres. Our results may be extrapolated to other regions with similar demographics and social contact patterns to those of Ontario.

The social structures of the population that were explicitly identified include households, schools, workplaces and public transportation. We also considered a generic social place to represent all other locations in the general community for related activities (e.g., shopping centres, public events). Individuals were present in, and travelled between, these social places according to *ad-hoc* schedules for daily activities. Because of computing time and memory constraints, we scaled down the modelled population size by a factor of 200 compared to the actual size of Ontario, but kept the same demographics distributions (see Supplementary Information).

The age distribution of the population was fitted to the Ontario data extracted from the 2011 Census^19^. Household compositions and school sizes were parameterized according to their respective distributions obtained from Statistic Canada^20^ and the Ontario Ministry of Education^21^. The size distribution of workplaces was fitted to data collected from Statistics Canada^22^. We parameterized the model for the entire province with databases available for the size and usage of public transportation from the Toronto Transit Commission data^23^.

#### Infection timelines

For the disease component, we used a standard representation of influenza natural history. Upon disease acquisition as a result of contact with infectious individuals, susceptible individuals enter a latent stage, during which they are not infectious. After the latent period has elapsed, infected individuals progress to the infectious stage, which can be either symptomatic or asymptomatic. The proportion of asymptomatic infections was set at 25% in the absence of vaccination^24^. The probability of developing symptomatic infection was determined based on the level of individual’s frailty, pre-existing cellular immunity and vaccine-induced protection at the individual level (see Supplementary Information). Infectious individuals may recover without any interventions. Antiviral treatment and hospitalization were considered only for symptomatic cases. Hospitalization of a symptomatic case was determined probabilistically based on the individual’s age-dependent frailty (see Supplementary Information). We used previous estimates related to the 2009 H1N1 pandemic to fit the time delay from the onset of symptoms to hospitalization^25^. Hospitalized cases either recovered or died from influenza infection with an age-dependent probability of death^26^. The durations of latency, infectiousness and hospitalization for each individual were drawn from lognormal distributions with means of 1.5, 3.0 and 12 days respectively^25,27–29^ (see Table 1 and Supplementary Information).

**Table 1:** Model parameters for the scenarios VPS1, VPS2 and RVS.

Baseline Scenario	Antiviral treatment	Self-isolation	Transmissibility (*R*_*0*_)
Priority age group	10% of symptomatic cases	90% of symptomatic cases	moderate (R0 = 1.4) high (R0 = 1.9)
**Vaccination Scenarios**	**VPS1**	**VPS2**	**RVS**
Priority age group	0–4 and 65+	0–18 and 65+	all individuals (no specific priority)
Priority frailty	above the average frailty of the population		all individuals (no specific priority)
Maximum vaccine coverage for the entire population	40%		
Vaccine efficacy	high (max. 90%) low (max. 40%)		
Vaccination time lag (weeks to outbreak onset)	−4; −2; 0; 2; 4		
Vaccine doses administered per day (per 100,000 population)	150; 300; 600; 1200		
**Disease specific main parameters**	**Distribution**	**Mean (variance)**	
Latent period (days)	Log-normal	1.25 (0.1)	
Infectious period (days)	Log-normal	3.0 (0.4)	
Hospitalization duration (days)	Log-normal	12 (21)	
Asymptomatic fraction	—	0.25	
Asymptomatic relative infectiousness	—	0.1	
**Age groups fraction of the population**			
**0–4**	**5–18**	**19–64**	**65+**
4.5%	17.0%	63.8%	14.8%

#### Individual attributes

We assumed no pre-existing humoral immunity in the population at the time of pandemic emergence. The pre-existing cellular immunity was included in the model with the effect of reducing the probability of developing clinical symptoms, if infected. This effect was parameterized for different age groups based on estimates for the 2009 H1N1 pandemic^30^ (see Supplementary Information). We also considered a frailty index, which was modelled as an age-dependent parameter and fitted to the data (extracted from the 2014 Canadian Community Health Survey) for the proportion of individuals in different age groups with a major chronic disease and risks of hospitalization and death for seasonal influenza. The frailty index at the individual level was used to determine the vaccine efficacy and the probabilities (conditional upon infection) to develop symptoms, become hospitalized, or die as an outcome of infection (see Supplementary Information).

#### Transmission dynamics and transmissibility

Transmission between infectious and susceptible individuals was successful based on the outcomes of rejection sampling trials where the chance of success is defined by a transmission probability distribution. The transmission dynamics relied on individuals’ movements between social places and their daily contact rate (i.e., the number of contacts per day). The age-dependent contact rates followed a lognormal distribution to allow for super-spreading events^31^. We used two sources of information to fit age-dependent contact rates, including a retrospective study of the timing of symptoms onset stratified by age across several influenza seasons in Canada^32^, and face-to-face contact studies in France^33,34^. The main difference between the two sources is that the former estimates the largest effective contact rates for teenagers and young adults, while the latter suggest that young children have the highest contact rates. We fitted the age component of contact rates from the Canadian retrospective study in our main simulations, and investigated the differences arising when using the face-to-face contact data (Figure S6, Supplementary Information). These values were calibrated to arbitrarily chosen basic reproduction numbers within the estimated ranges for previous pandemics^35^. The basic reproduction number (denoted by *R*_0_) is a theoretical quantity defined as the average number of secondary infections generated by the introduction of a single infectious individual in a fully susceptible population^36^. This quantity is commonly used to measure the transmissibility of a disease and the effect of intervention measures. We explored both moderate (*R*_0_ = 1.4) and high (*R*_0_ = 1.9) reproduction numbers for pandemic outbreaks simulated in the baseline scenario without vaccination.

### Interventions

We modelled antiviral treatment and pandemic strain-matched vaccination as pharmacological interventions. For other community measures, we only considered self-isolation with the probability of 90% for symptomatic cases only^37^. We assumed that antiviral treatment is offered to only 10% of symptomatic patients^38^ with the effect of reducing infectiousness by 10% and the duration of infectious period by 1 day on average^3^. We assumed a 0.5-day delay in start of antiviral treatment after the onset of symptoms for those who sought care. Antiviral drugs had no effect if taken 2.5 days after symptoms onset.

To implement vaccination, we considered different timelines for vaccine availability including the possibility to start vaccination before the onset of pandemic outbreak. We defined the vaccination time-lag as the difference between the start date of the vaccination campaign and the first pandemic case in our simulations. Thus, a negative time-lag corresponds to a vaccination campaign starting *before* the onset of the outbreak. We explored time-lag values of −4, −2, 0, +2 and +4 weeks (Table 1). The rate of vaccine administration for seasonal influenza is estimated at about 0.3% of the population per day (or 300 vaccine doses per 100,000 individuals per day)^39^. In our study, we investigated administration rates of 150, 300, 600 and 1,200 vaccine doses per 100,000 individuals per day, with the two highest rates being above the current estimates. All vaccination scenarios assumed a single vaccine dose.

No large randomized clinical trials of pandemic strain-matched vaccine efficacy have been performed in human populations yet. However, several meta-analysis estimated the effectiveness of strain-specific monovalent vaccines after the 2009 H1N1 pandemic and found high vaccine effectiveness in preventing laboratory-confirmed influenza, with point estimates ranging from 69% to 90%^40–42^. Moreover, some new vaccine manufacturing technologies (e.g., insect cell-derived recombinant vaccine, virus-like particle) show no risk of mutation associated with manufacturing in eggs that can affect vaccine effectiveness^43^. We therefore considered two vaccine efficacies with a maximum probability of 90% (“high efficacy”) and 40% (“low efficacy”) for preventing infection in immunocompetent individuals. The vaccine-induced protection efficacy at the individual level was determined probabilistically, and reduced from the maximum level according to the sampled frailty index (i.e., the vaccine efficacy reduces as the frailty increases, see Supplementary Information). When vaccination was successful in full protection, we assumed a linear increase in the vaccine-induced protection over a two-week period following vaccination. We also assumed that vaccination decreased the probability of developing symptomatic infection, if infected^44,45^. Further details of the vaccine implementation in the model are provided in the Supplementary Information.

### Vaccination strategies

We first considered a vaccine prioritization strategy (“VPS1”) that largely aligns with the recommendation outlined in the Ontario Strategic Influenza Pandemic Planning, which follows closely the recommendations from the Public Health Agency of Canada^46^. Initial vaccine distribution in the VPS1 was implemented for priority groups including children under 5 years of age; individuals 65+ years of age, and other individuals (of any age) with a frailty index higher than the average frailty of the entire population. When the coverage of these priority groups reached the reported average of 34.5% (which was the average vaccine uptake for Ontario during the 2009 H1N1 pandemic)^39^, vaccination of other age groups was also initiated. To evaluate the effect of vaccinating school children, we simulated another vaccine prioritization strategy (“VPS2”) where the 6–18 age group was also included in the list of priority groups of the VPS1. For comparison purposes, we implemented a random vaccination strategy (“RVS”), without any prioritization. All three strategies have a maximum vaccination coverage set at 40% of the entire population.

### Model calibration

We calibrated the model with Ontario demographics data (Figure S1). We used hospitalizations and deaths rates of 90 and 20 per 100,000, respectively, which are representative of more severe pandemic strains based on historical records^26^ (Figure S10). The transmissibility of the pandemic strain was calibrated to the given reproduction number for each scenario. We ran 500 Monte-Carlo iterations for each scenario and considered the average of sample realizations, while omitting simulations where the baseline scenario with no vaccination had a final cumulative incidence lower than 1% of the total population. Main outcome measures in scenario evaluation were the relative reductions in clinical attack rates, hospitalizations and deaths compared to the baseline scenario that only includes antiviral treatment without vaccination.

## Results

The relative reductions of the clinical attack rate (CAR), hospitalizations and deaths were evaluated in a number of scenarios outlined in Table 1, which combine different values of vaccine efficacy, pandemic transmissibility, time-lag for the start of vaccination, and the rate of vaccine administration.

### Relative reduction of the overall clinical attack rate

**Figure 1:**
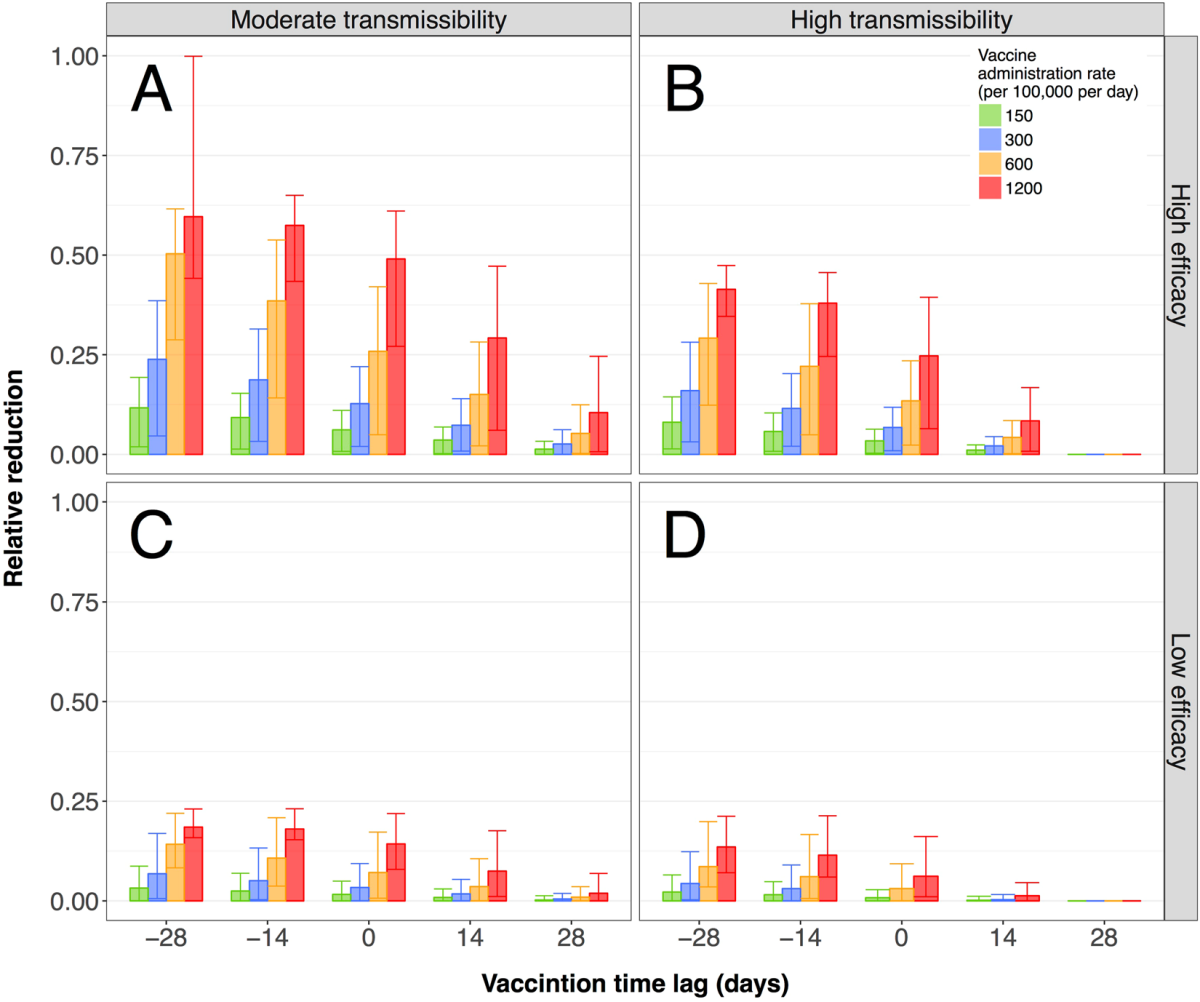
Relative reduction of overall clinical attack rates. For a given transmissibility and vaccine efficacy scenario, each panel represents the relative reduction of the clinical attack rates when compared with the baseline scenario without vaccination. The bars represent the mean and the whiskers show the 5% and 95% quantiles of the relative reductions. The vaccination time lag is represented on the x-axis. For each time lag, the relative reductions for a given vaccine administration rate is shown as grouped coloured bars. All panels show results under the VPS1 strategy and age-specific contact data calibrated to the Canadian retrospective study^32^.

For a scenario where the pandemic strain has a moderate transmissibility (*R*_0_ = 1.4), an early vaccination campaign coupled with a highly efficacious vaccine and a sustained vaccine administration rate has the potential to markedly reduce the CAR compared with the baseline scenario (i.e., no vaccination). For example, as shown in Fig. 1A, if the VPS1 started with the time-lag of −4 weeks, we observed a mean relative reduction of 24% (90%CI: 5–38%) for the CAR with the administration rate of 300 vaccine doses per 100,000 population per day. The CAR reduced by 60% (90%CI: 44–100%) when the administration rate was increased by 4-fold to 1,200 vaccine doses per 100,000 population per day. In this scenario, the upper bound value of the 90% quantile at 1 suggests the possibility to avoid a large outbreak. However, when the time-lag is positive (i.e., vaccination starts after the onset of the outbreak), the mean relative reduction of the CAR is significantly reduced to below 50% even when a highly efficacious vaccine is offered at a high administration rate (Fig. 1A). Simulation results for VPS1 with a highly transmissible pandemic strain (*R*_0_ = 1.9) indicate lower mean reduction of the CAR below 40% in the corresponding scenarios (Fig. 1B).

For a low-efficacy vaccine, the mean relative reduction of the CAR is substantially decreased to below 20% for all the scenarios regardless of the time for start of vaccination (Fig. 1C,D). Compared with the moderate transmissibility, our results indicate that averting large outbreaks is not feasible.

Further simulations under the VPS2 indicate that the additional benefits of including school children in priority groups for vaccination in the VPS1 were marginal in terms of reducing the CAR for all the corresponding scenarios (Figure S5).

When age-contact structures in our model were modified based on data from the face-to-face contact studies^33,34^, we estimated larger relative reductions compared with the corresponding scenarios described above using the Canadian retrospective study^32^. However, the overall reduction pattern remained similar between the two contact structures (Figure S6). Finally, we observed a similar reduction pattern in the attack rates of both symptomatic and asymptomatic infections.

### Relative reduction of hospitalizations and deaths

**Figure 2:**
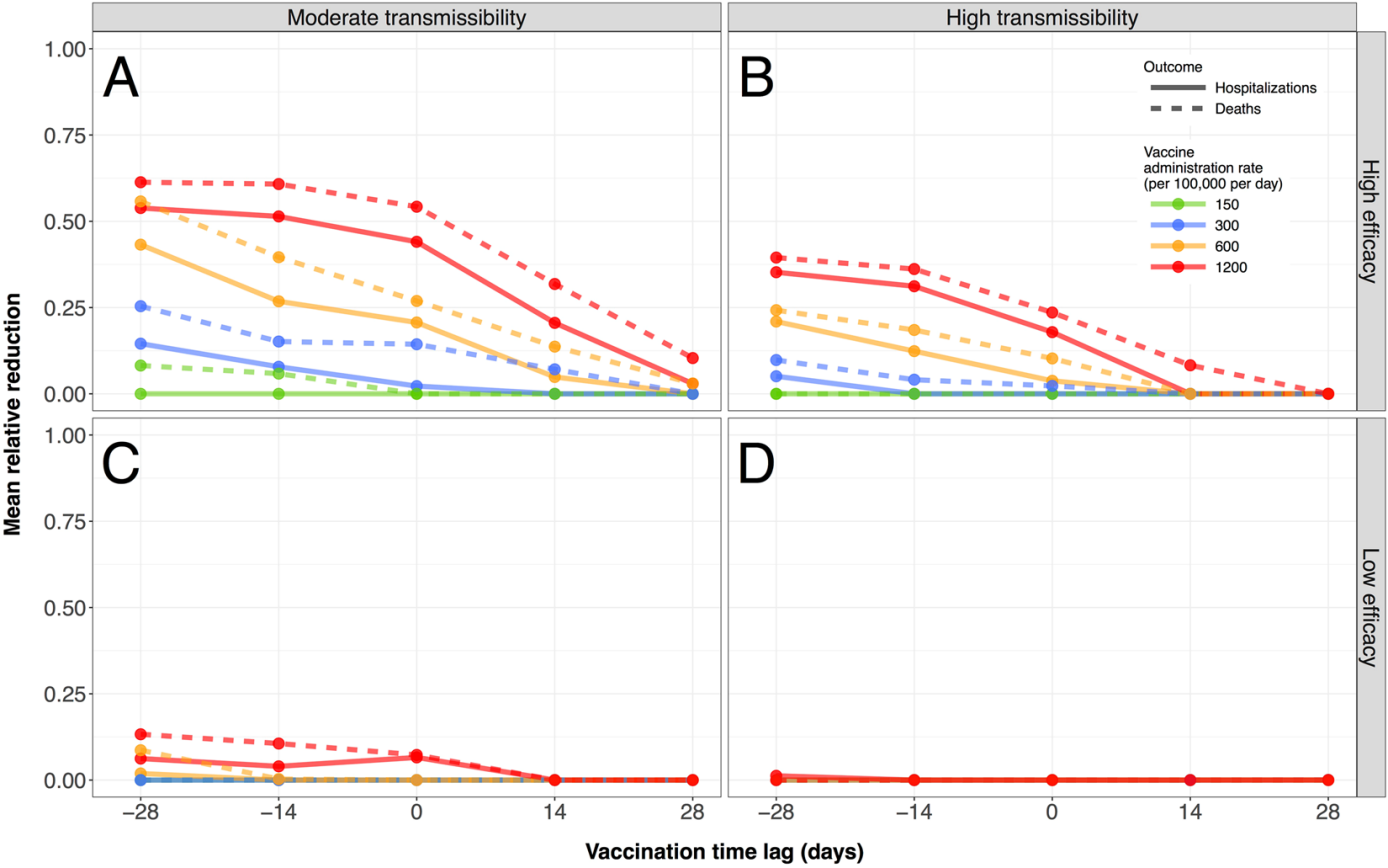
Relative reduction of deaths and hospitalizations. For a given transmissibility and vaccine efficacy scenario, each panel represents the mean relative reduction of the overall deaths (solid lines) and hospitalizations (dashed line) when compared with the baseline scenario without vaccination. The vaccination time lag is represented on the x-axis. All panels show results under the VPS1 strategy and age-specific contact data calibrated to the Canadian retrospective study^32^.

For a high-efficacy vaccine, VPS1 could lead to a substantial reduction of hospitalizations and deaths when vaccination started before the outbreak (Fig. 2). The mean relative reductions of hospitalizations and deaths achieved in the moderate transmissibility scenarios were estimated in the range 50–60% (Fig. 2A), which correspond to the start of vaccination four weeks before the outbreak with a high administration rate. When considering vaccine administration rate at the average rate for seasonal influenza (i.e., 300 doses per 100,000 population per day), this maximum reduction fell to 25% for deaths and 15% for hospitalization. A similar pattern emerged in high transmissibility scenarios, with smaller reductions of hospitalizations and deaths that remain under 40% (Fig. 2B).

Simulating the VPS1 under the scenario of a low-efficacy vaccine, we found little (<15%) or no reduction of hospitalizations or deaths compared with the baseline scenario (Fig. 2C,D). Overall, we observed a higher uncertainty in the results for hospitalizations and deaths, as these are relatively rare events compared with disease transmission.

Reductions of hospitalizations and deaths under the VPS2 were similar (with insignificant difference) to those observed under the VPS1 in the corresponding scenarios, whether the age-specific contact structure was calibrated to the retrospective study^32^ (Figure S7) or the face-to-face contact data^33,34^ (Figure S8).

### Comparison between priority and random vaccination strategies

We ran simulations with the RVS and compared the population-wide and age-specific outcomes of the CAR, hospitalizations, and deaths with those obtained using VPS1 and VPS2 (Figures S3, S4). The mean relative reductions were slightly higher in both VPS1 and VPS2, suggesting the potential for achieving better outcomes by prioritizing high-risk and highly interactive individuals. Overall, we observed a similar trend in the relative reductions between these strategies across all scenarios when vaccine efficacy, disease transmissibility, and timelines for the start of vaccination were varied. These observations also hold true when the contact structure of the population was shifted from the Canadian retrospective study^32^ to resemble those obtained in the face-to-face contact studies^33,34^.

### Sensitivity analyses

We relied on a number of estimates related to the 2009 H1N1 pandemic and seasonal epidemics (see SI Table S1). However, the nature of a future pandemic cannot be predicted with certainty. We therefore performed a sensitivity analysis to account for possible variations in a number of model parameters including the infectiousness ratio for asymptomatic cases, the age-assortativity contact structure, the contact rate coefficient of variation, and the frailty standard deviation at the individual level. Results from this sensitivity analysis do not alter our interpretation of the results, indicating the robustness of the model outcomes (Supplementary Information, Figure S9).

## Discussion

Our study, using an agent-based computational approach, quantified the epidemiological impact of a vaccination campaign against an influenza pandemic in the province of Ontario, Canada, under plausible scenarios. In particular, given recent advances in influenza vaccine manufacturing, we investigated the effect of the timing and speed with which the vaccination roll-out is implemented.

Similar to a previous study^7^ for the entire population of the United States, we found that the morbidity and mortality reductions are mainly determined by the timing for the start of the vaccination campaign and the rate of vaccine administration. Although a direct comparison of results is delicate, our model tends to estimate a larger proportion of cases averted, especially for scenarios where vaccination starts early (e.g., 4 weeks before the outbreak in scenario VPS1). Such a difference may be in part due to distinct modelling approaches. For example, our study uses a dynamic and adaptable simulation method, and therefore takes into account the indirect benefits of vaccination such as herd immunity. The results in the US study may be conservative given the more static nature of its mathematical model. Higher vaccine-induced reductions of the clinical attack rate were projected in other settings. In the urban population of Vancouver, Canada, a simulation study^6^ found a reduction of about 80% for both morbidity and mortality when vaccination started 2 weeks before the epidemic onset, with an average administration rate of 900 vaccine doses per 100,000 population per day, and a moderate transmissibility (*R*_0_ = 1.4). Our simulations found approximately a lower (Fig. 2A) reduction for a similar range of parameters in the context of Ontario.

Using data from retrospective and behavioural studies, young adults or children effectively acted as a core transmission group in our simulations. In an exploratory analysis, we found that strategies that excluded such groups from vaccination would always lead to a small overall vaccination impact. Although children were not specifically targeted in the RVS, this strategy vaccinated enough of them such that the differences with VPS1 scenarios in terms of the relative reduction of the CAR, hospitalization and deaths were small. These results are consistent with previous studies, which highlighted the central role of school children in influenza transmission^2,47,48^.

We found that the contact structure of the population could have a considerable effect on the outcomes of vaccination strategies. Comparing Fig. 1 with Figure S6, we observed a modest increase of the vaccination impact when the age-specific contact patterns favoured large contact rates among children under 10 years of age (sourced from face-to-face contact data)^33,34^ compared to patterns where teenagers and young adults have the largest contact rates (sourced from a retrospective study of influenza symptoms onset timing)^32^. The impact of vaccine was higher using face-to-face contact data, especially for the VPS1 that prioritizes children less than 5 years of age. This difference in vaccine impact between these two parameterizations highlights the well-known sensitivity of mathematical models to contact patterns assumptions^2^. Moreover, using the two contact patterns in our simulations implicitly provides a measure of sensitivity for the results, indicating broadly similar levels and trends of vaccination effect in scenario outcomes (Figs 1 and S6).

The reduction in the clinical attack rate resulted from vaccination has also the indirect benefit of reducing the use of antiviral drugs. Because of the way antiviral treatment is implemented in our model (i.e., a fixed proportion of symptomatic individuals receive antiviral drugs), the relative reduction of antiviral drug usage is very similar to the reduction observed for symptomatic cases. Although not addressed here, lower antiviral drugs consumption as a result of vaccine effectiveness should be incorporated in future models for drug stockpiles and cost-effectiveness analyses. Another possible indirect benefit, although difficult to quantify, is the time delay in the importation of the first case and slower rate of disease spread due to early vaccination.

While we made our best efforts to simulate realistic pandemic scenarios, our study has several limitations. Because of the limited availability of data, population movements in Ontario were simplified without considering the geographical clustering. Also, public transportation was parameterized based on Toronto data only; hence our results may be more representative of urban centres. Our starting point for the vaccine administration rate was the value observed for seasonal influenza vaccination obtained from Statistics Canada^39^. We assumed that providers and health professional networks will be marshalled to deliver and administer vaccines for the early-vaccination strategies considered in our study. We have not encountered any reports on vaccine administration rates to be larger than those reported for seasonal influenza (e.g., 300 doses per 100,000 per day), and therefore reaching rates which are multiples of the baseline may require significant resources and planning by public health. There are two potential impediments that could slow down the administration rates including vaccine delivery by manufacturers and vaccine distribution by the healthcare system. Stockpiling vaccines may alleviate the logistical challenges associated with vaccine production and delivery by manufacturers. Since the nature of pandemic influenza viruses is unpredictable, our model cannot suggest any specific vaccine for stockpiling. However, as our results show, early availability is an important parameter in pandemic response. As vaccine production capacity improves and new technologies emerge with the regulatory framework adjusting to them^49^, it is expected that the manufacturing and delivery times will be reduced. These considerations, in addition to the characteristics of the potential pandemic strains, will need to be taken into account for decisions on vaccine stockpiling.

Further limitation of our study pertains to the assumption of continuous and constant supply of vaccine throughout the epidemic. In particular, scenarios in which vaccination is interrupted for a certain period of time or vaccine doses are administered at different rates over time (for a variety of reasons, e.g., manufacturing capacity, logistics or healthcare system delivery) were not considered here and might alter our findings.

Despite the possibility that a pandemic vaccine may require two doses to provide protective levels of immunity^50^, we considered only single-dose vaccination scenarios in addition to antiviral treatment. We however note that the inclusion of two-dose vaccination involves additional assumptions and unknown parameters on the vaccine efficacy for each dose, and the time interval between first and second doses of vaccine. Furthermore, a two-dose strategy must contend with the possible drop-out, whether due to acquiring infection after the first dose or simply due to individuals voluntarily forgoing the second dose. These factors may in turn increase the uncertainty of model outcomes. However, the dynamics of two-dose vaccination scenarios may be accounted for by considering a slower administration rate and a range of vaccine efficacy in a single-dose vaccination programs.

For the vaccination coverage, we parameterized our model with a baseline value of 40% reported for seasonal influenza in Ontario, Canada. We note that our results are relatively insensitive to higher values of vaccine coverage, because the vaccination rates used in our scenarios (also based on the rates observed during seasonal influenza) rarely reached sufficiently high values early enough in the epidemic to have an impact on disease outcomes in terms of hospitalization or deaths.

Given the range of possibilities, the outcomes of different scenarios in reducing the overall disease burden cannot be accurately quantified. Furthermore, data on human behaviour (relevant to influenza transmission) is generally scarce, and even more so when considering a specific population setting. The difference in vaccination strategy outcomes between the age-specific contact parameterizations using retrospective and face-to-face contact data (Figs 1 and S6) shows the sensitivity of the results to these assumptions. These factors highlight the need for population specific parameterizations in future models to more accurately quantify the outcomes.

## Conclusion

Early vaccine availability with a high administration rate has the potential to substantially reduce the clinical attack rate, hospitalizations, and deaths. Vaccine administration at a rate of 0.3% of the population per day for seasonal influenza (as the baseline) would still provide modest reductions if vaccination campaigns are initiated four weeks before the outbreak. If vaccine administration occurs at only half that rate (i.e., 0.15% per day), our simulations suggest that the benefits of early vaccine availability may be very limited, even in scenarios with a highly efficacious vaccine and moderate transmissibility (Fig. 1). In other words, a slow rate of vaccine administration can potentially offset the benefits of early vaccine availability. This highlights the importance of investment in both manufacturing and healthcare capacities in planning for pandemic vaccination. The findings suggest that vaccination strategies that prioritize high-risk individuals and young children have the largest impact in terms of reducing the morbidity, mortality, and severe outcomes (i.e., hospitalization). Although contact patterns of the population can influence the outcomes, the conclusion on strategy effectiveness remains intact.

### Availability of data and material

Parameterization using available data is detailed in the online Supplementary Information. The source code to reproduce the results is available by contacting the corresponding author.

## Electronic supplementary material

Supplementary Information
