# Supplementary material for: Assessing the benefits of early pandemic influenza vaccine availability: a case study for Ontario, Canada
Source: Sci Rep. 2018 Apr 24;8:6492. doi: 10.1038/s41598-018-24764-7 (PMC5915538; doi:10.1038/s41598-018-24764-7)
Supplement: Supplementary file 1 — Supplementary Information [file 41598_2018_24764_MOESM1_ESM.pdf]

Supplementary Information

Assessing the benefits of early pandemic influenza vaccine availability: a case study for Ontario, Canada

David Champredon[1,], Marek Laskowski[1], Nathalie Charland[2], Seyed M. Moghadas[1]*

[1] Agent-Based Modelling Laboratory, York University, Toronto, M3J 1P3 Ontario, Canada

[2] Medicago Inc., 1020 Route de l'Eglise, Quebec, G1V 3V9 Quebec, Canada

* Corresponding author: david.champredon@gmail.com

Contents:

- Supplementary figures
- Model description

Figure S1 – Model fit on demographic data.

Black crosses show the model outputs and red circles the data used for the model fit. Left panel shows the age distribution of the population for the province of Ontario, Canada. Right panel shows the household size distribution for the same region (households larger than six were censored). Data is from Statistics Canada's 2011 Census for both panels.

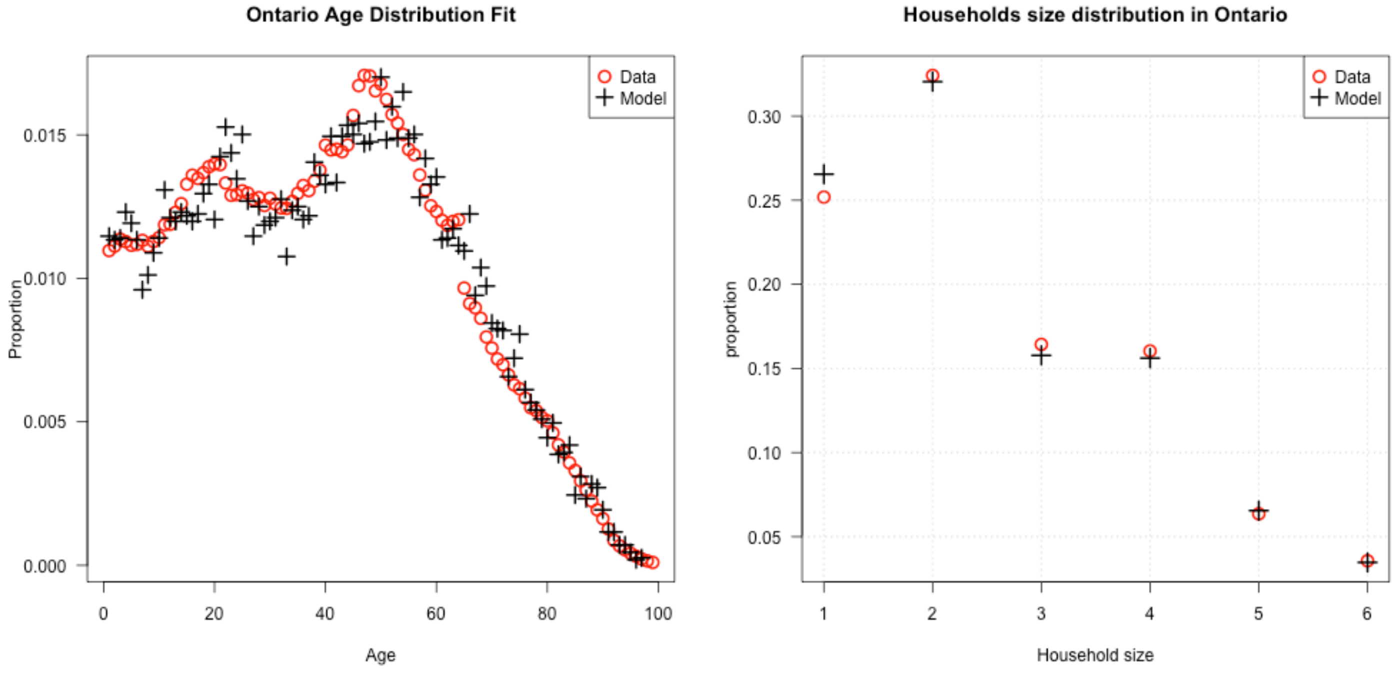

Figure S3 – Comparing vaccination strategies impact on the relative reduction of overall deaths, hospitalizations and clinical attack rates.

Points represent the mean relative reduction of the relevant outcome when compared with the same scenario without vaccination. Each column represents a combination of vaccine efficacy and pandemic transmissibility. Each row represents a different outcome (first row deaths, second row hospitalizations and last row clinical attack rates). Solid lines represent the strategy PVS1 and the dashed line RVS. In all panels, none of the paired curves difference is statistically significant (the credible intervals [not shown] overlap). All panels show results with age-specific contact data calibrated on a Canadian retrospective study [32].

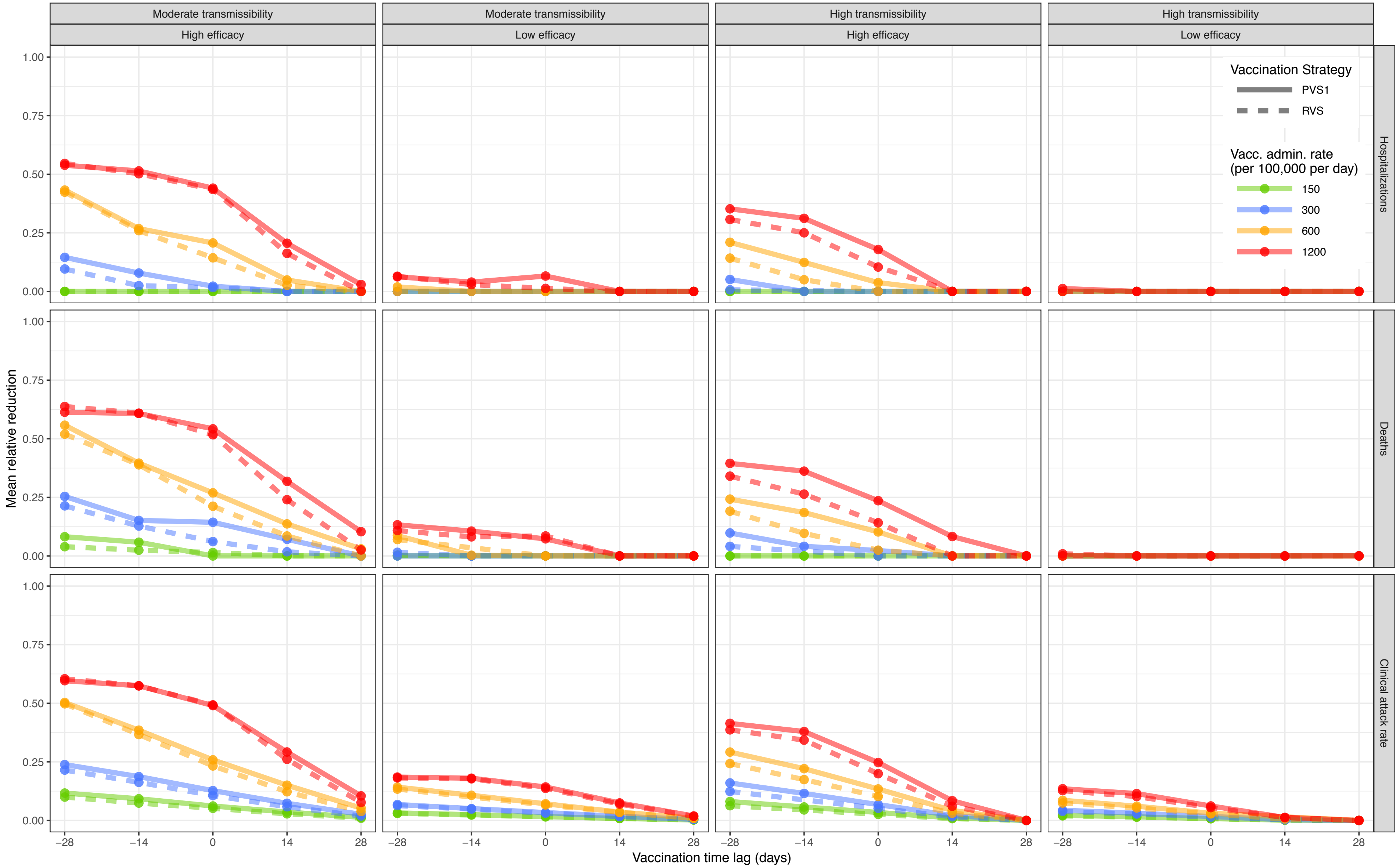

Figure S4 - Relative reduction of the age-specific clinical attack rates.

All panels show the mean relative reduction in age-specific clinical attack rates for vaccination strategies RVS (top half) and PVS1 (bottom half). We observed, as expected, a higher reduction among the under 5 group than the 5-18 one for PVS1 strategy.

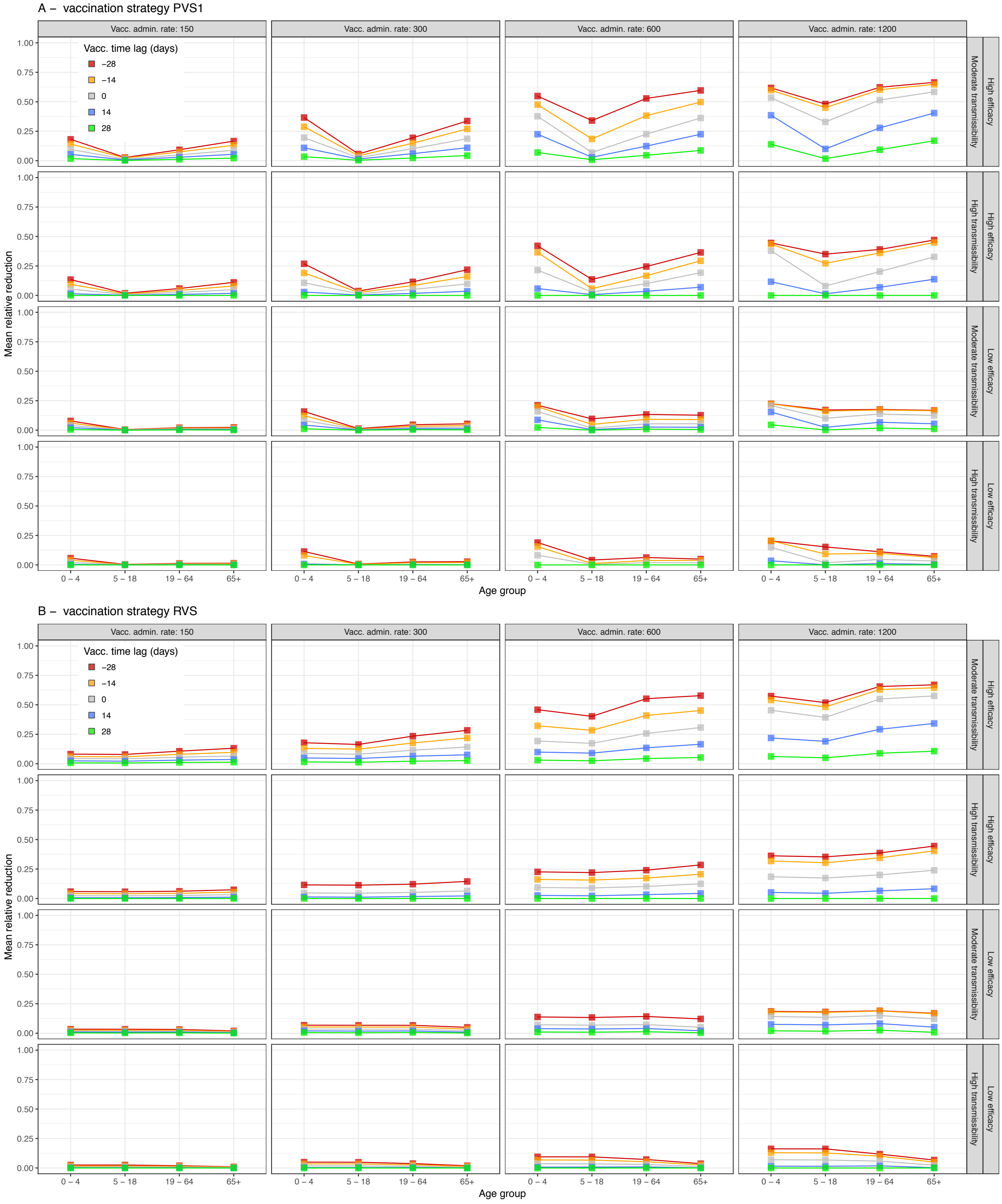

Figure S5 – Relative reduction of overall clinical attack rates with vaccination strategy PVS2.

Same legends as Figure 1, except that all panels show results under the vaccination strategy PVS2.

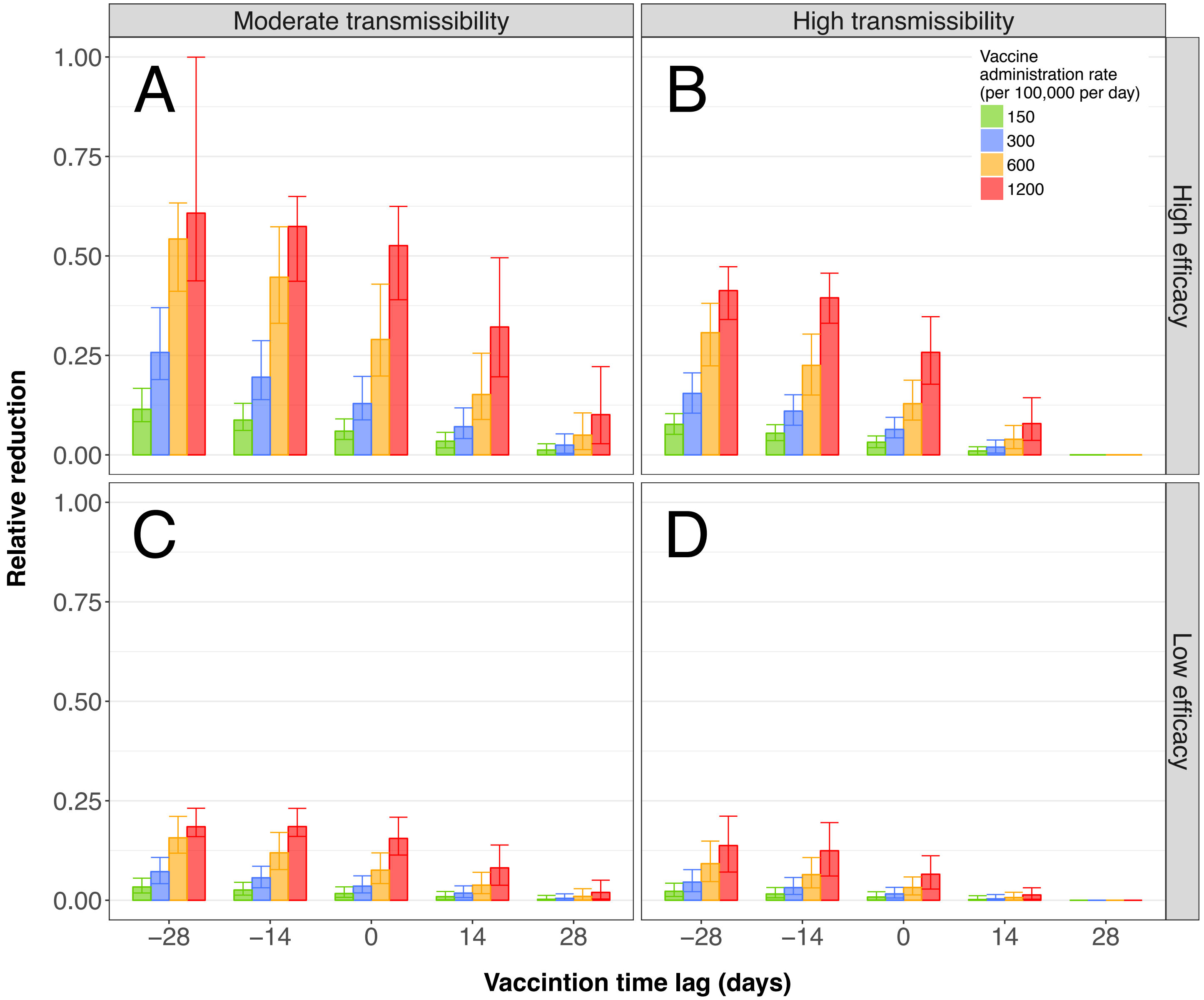

Figure S6 – Relative reduction of overall clinical attack rates with alternative age-specific contact rate parameterization.

Same legends as Figure 1, except that all panels show results under age-specific contact data calibrated on a French face-to face contact [33,34] (as opposed to data sourced from a Canadian retrospective study [32]).

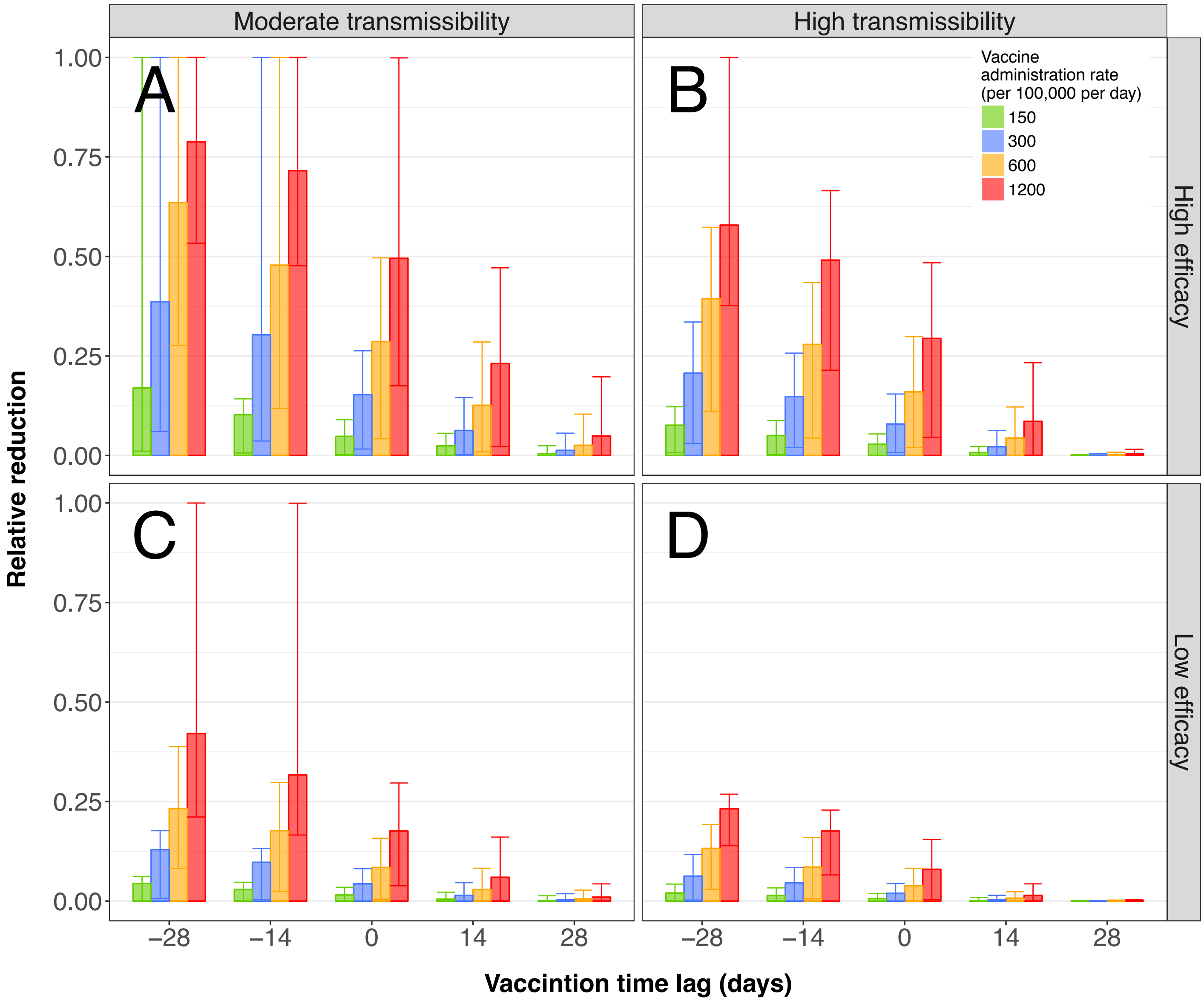

Figure S7 – Relative reduction of deaths and hospitalizations.

Each panel represents, for a given transmissibility and vaccine efficacy scenario, the mean relative reduction of the overall deaths (solid lines) and hospitalizations (dashed line) when compared with a scenario without vaccination. The vaccination time lag is represented on the x-axis. All panels show results under the PVS2 strategy and age-specific contact data calibrated on a Canadian retrospective study [32].

(The difference between Figure S7 and Figure 2 is that the former was run with strategy PVS2 and the latter with PVS1)

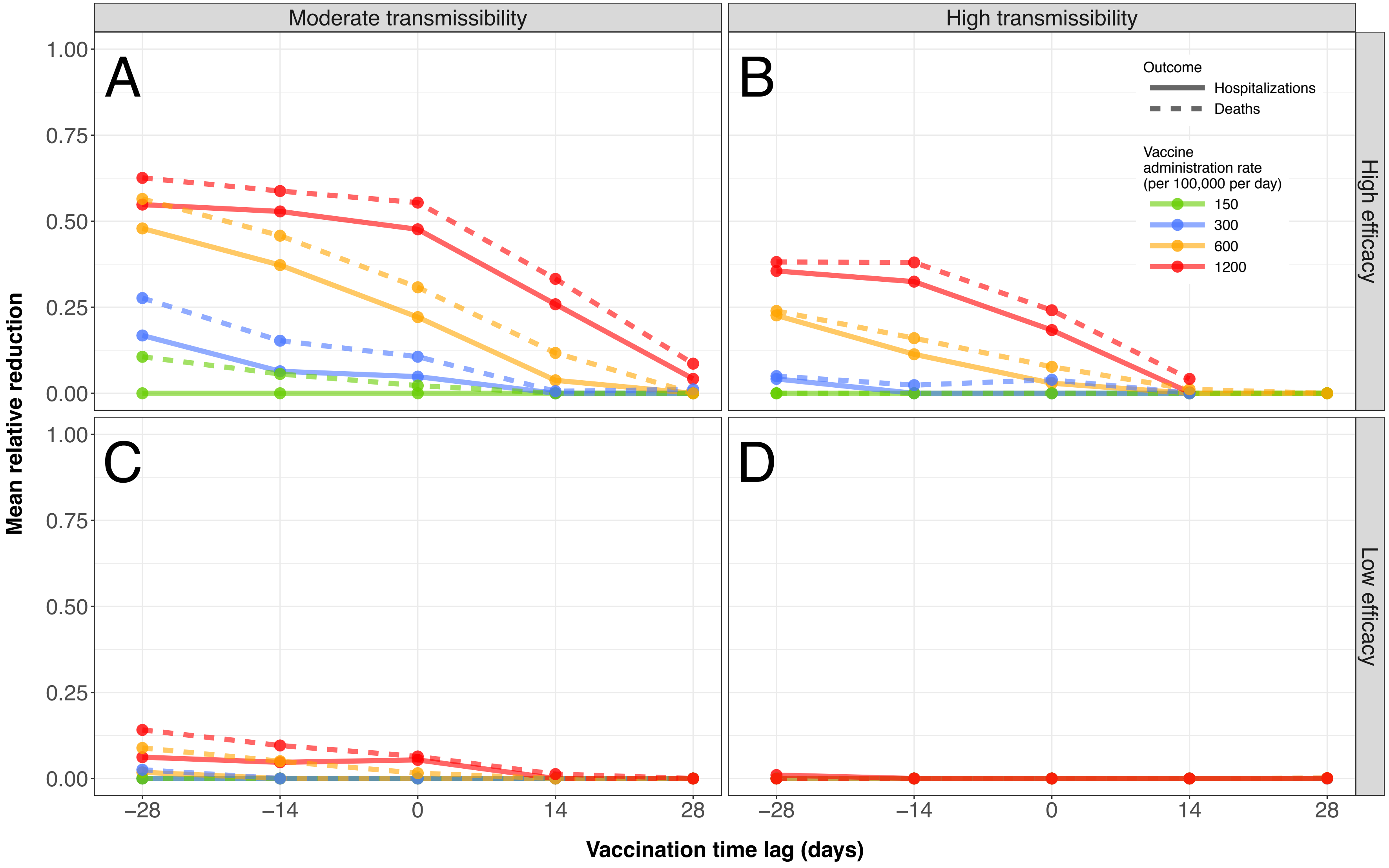

Figure S8 – Relative reduction of deaths and hospitalizations.

Each panel represents, for a given transmissibility and vaccine efficacy scenario, the mean relative reduction of the overall deaths (solid lines) and hospitalizations (dashed line) when compared with a scenario without vaccination. The vaccination time lag is represented on the x-axis. All panels show results under the PVS1 strategy and age-specific contact data calibrated on face-to-face contact studies [33,34].

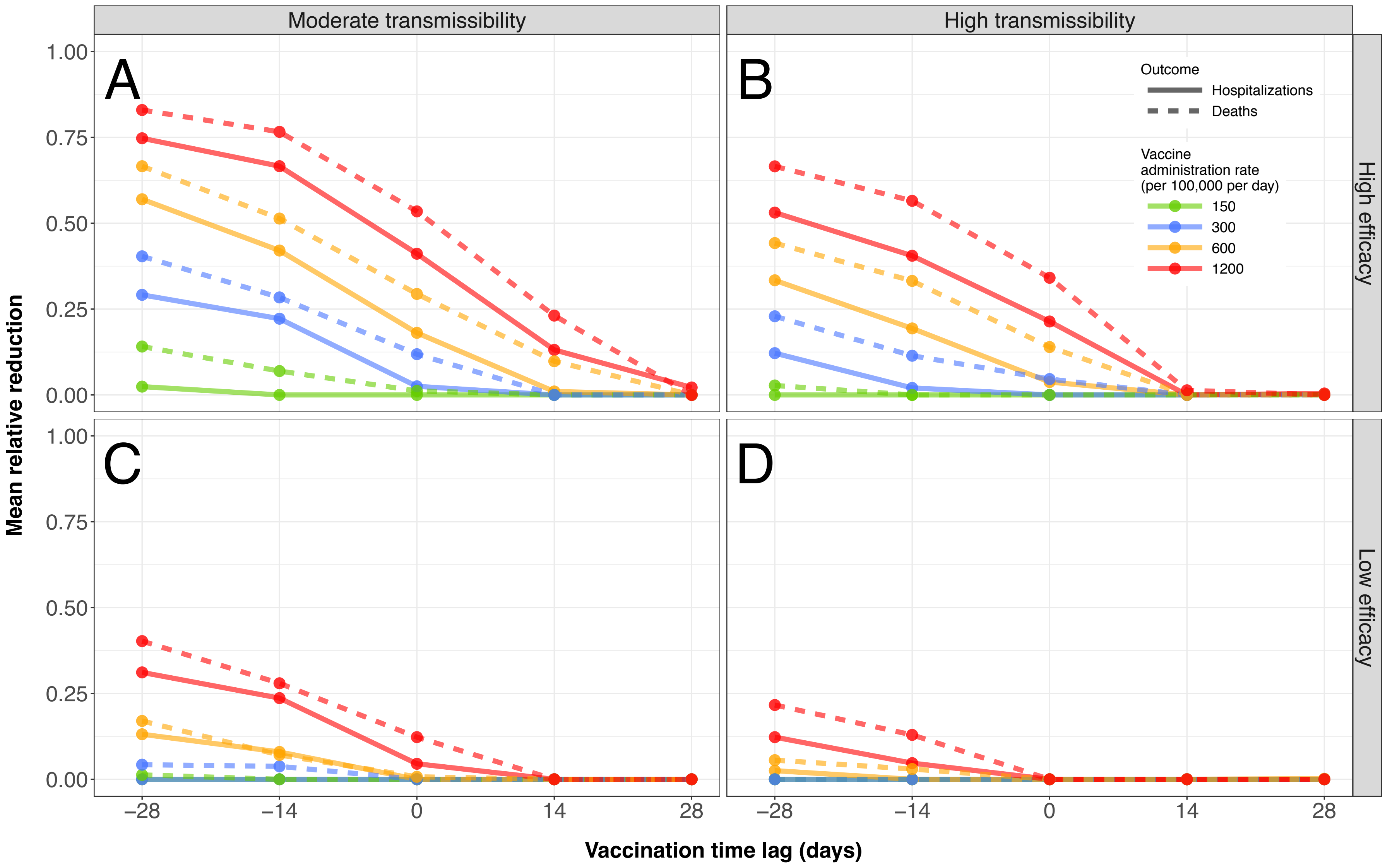

Figure S9 – Sensitivity Analysis.

A sensitivity analysis was performed on five model parameters. For each panel, the y-axis represents the range of mean relative reduction change for CAR when the associated parameter value was varied. The vaccination lag (-28 and +14 days) is represented by the x-axis. The bar fill color represents the vaccine administration rate. Each row is associated with one of the five parameters. The left column displays results for a 40% vaccine efficacy, the right column for a 90% efficacy. First row: the infectiousness ratio for asymptomatic cases was varied between 0.05 and 0.4 (baseline value was 0.1). Second row: contact age assortativity parameter was varied between 0.05 and 0.2 (baseline value was 0.1). Third row: contact rate coefficient of variation was varied between 0.3 and 1.5 (baseline value was 0.75). Fourth row: frailty standard deviation parameter was varied between 0.02 and 0.2 (baseline value was 0.1). Fifth row: maximum level of cellular immunity index was varied between 0.1 and 0.9 (baseline value was 0.45).

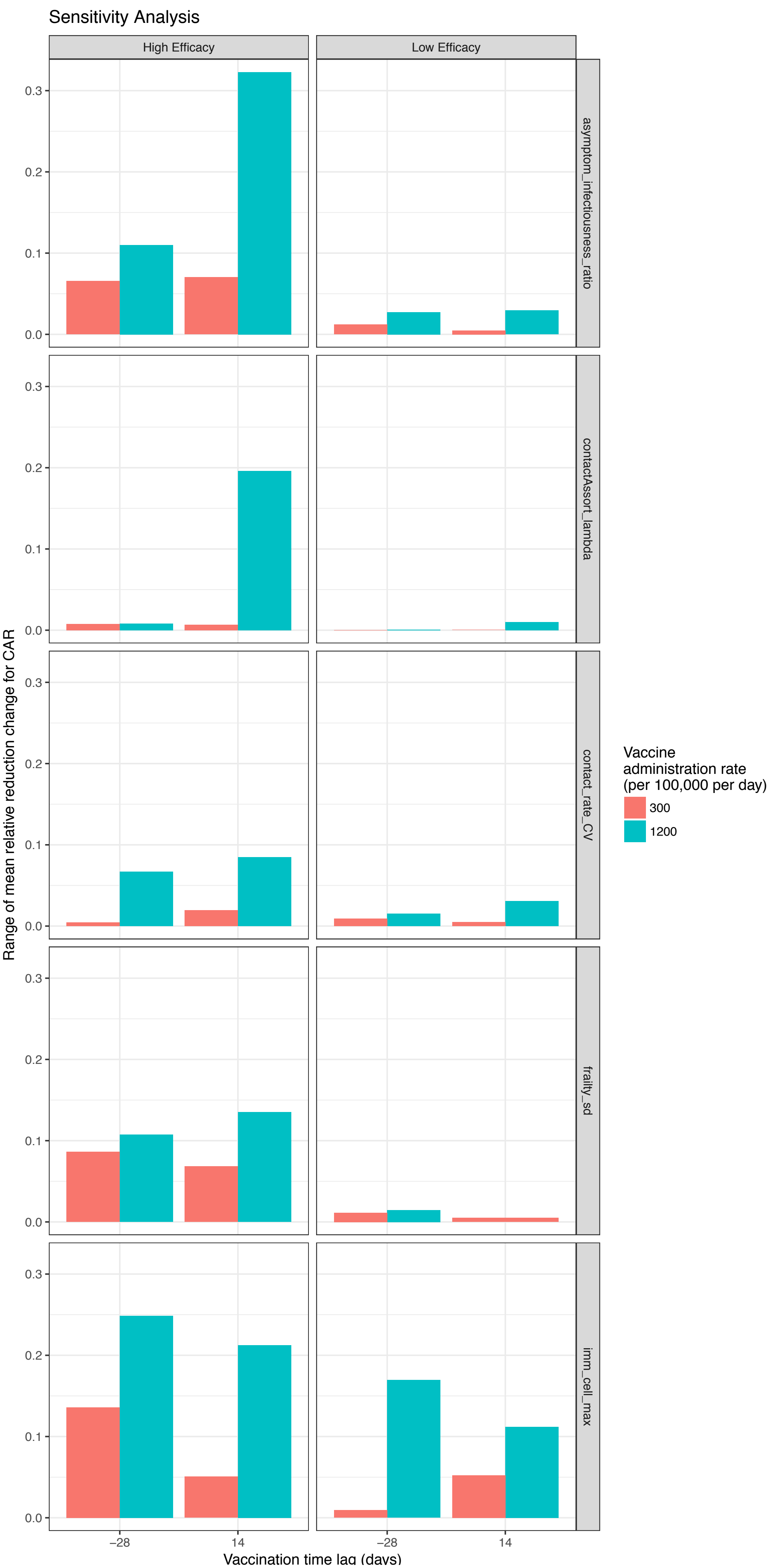

Sensitivity Analysis
High Efficacy
Low Efficacy
asymptom_infectiousness_ratio
contactAssort_lambda
contact_rate_CV
frailty_sd
imm_cell_max
0.0
0.1
0.2
0.3
−28
14
Range of mean relative reduction change for CAR
Vaccination time lag (days)
Vaccine
administration rate
(per 100,000 per day)
300
1200

Figure S10

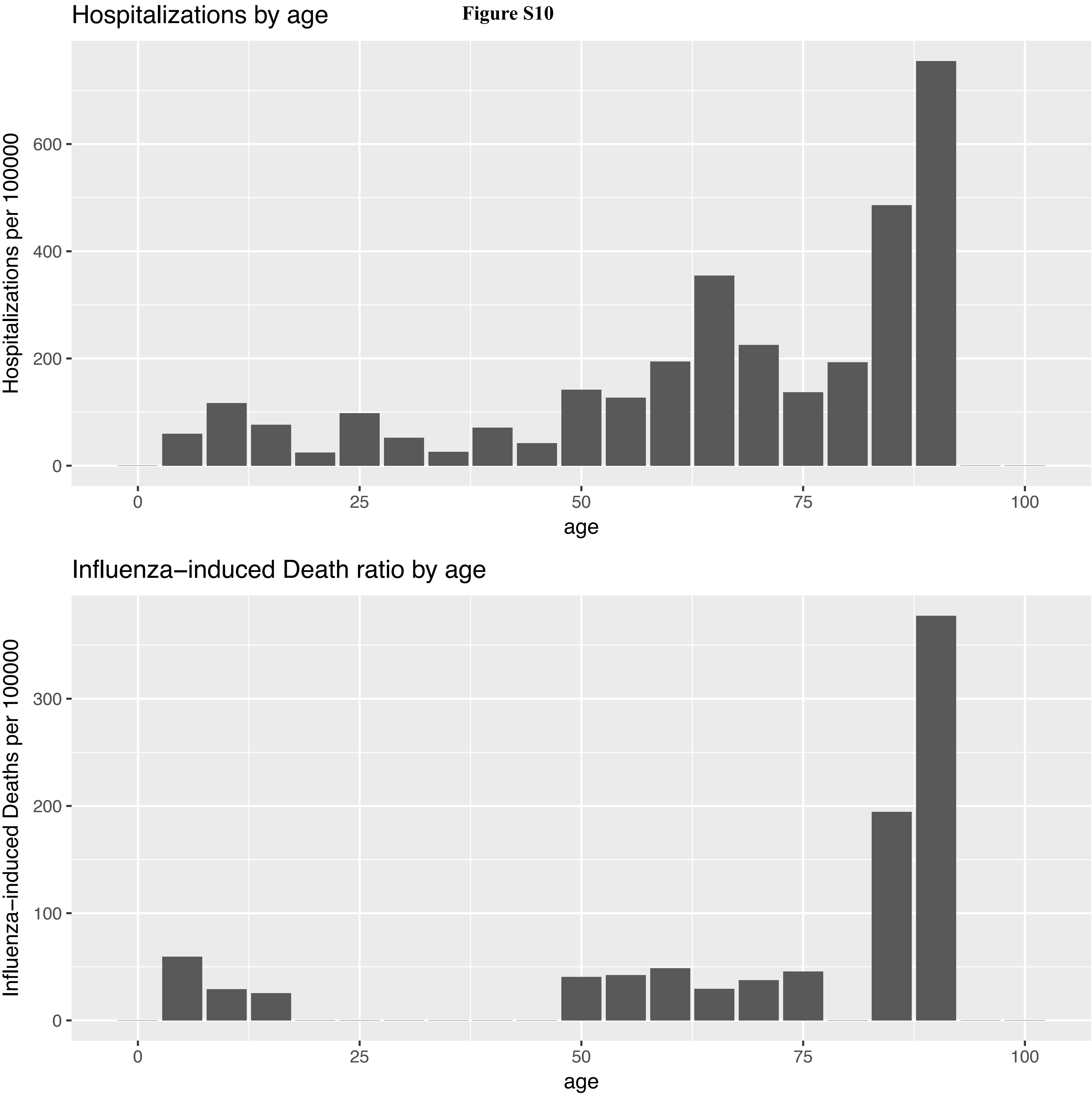
Hospitalizations by age
Hospitalizations per 100000
0
200
400
600
0
25
50
75
100
age
Influenza−induced Death ratio by age
Influenza−induced Deaths per 100000
0
100
200
300
0
25
50
75
100
age

Supplemental Material

Assessing the benefits of early pandemic influenza vaccine availability: a case study for Ontario, Canada.

David Champredon,[1,*] Marek Laskowski[1], Nathalie Charland[2], Seyed Moghadas[1]

[1] Agent-based modelling laboratory. York University, 4700 Keele Street, Toronto, Canada.

[2] Medicago Inc., 1020 Route de l'Église, Bureau 600, Quebec, G1V 3V9 Quebec, Canada

[*] Corresponding author: david.champredon@gmail.com

Contents

1 Overview **2**

2 Population **2**
- 2.1 Social place . . . 2
- 2.2 Individuals . . . 3
- 2.3 *in-silico* Population construction . . . 3

3 Simulator **5**
- 3.1 Model structure . . . 5
- 3.2 Schedules and spatial movements . . . 6
- 3.3 Frailty . . . 7
- 3.4 Humoral immunity . . . 8
- 3.5 Cellular immunity . . . 8
- 3.6 Transmission process . . . 9
  - 3.6.1 Number of contacts . . . 9
  - 3.6.2 Contacts selection . . . 10
  - 3.6.3 Transmission probabilities . . . 11
- 3.7 Disease progression . . . 12
  - 3.7.1 Asymptomatic and symptomatic infections . . . 12
  - 3.7.2 Hospitalization and death . . . 12

4 Interventions **13**
- 4.1 Deployment . . . 13
- 4.2 Antiviral treatment . . . 14
- 4.3 Vaccination . . . 15
  - 4.3.1 Effects of vaccine on disease transmission . . . 15
  - 4.3.2 Vaccination strategy . . . 16
  - 4.3.3 Vaccine administration rate . . . 17

5 Table of main model parameters **17**

This supplemental material describes the details and implementation of an agent-based model used for the article *"Assessing the benefits of early pandemic influenza vaccination: a case study for Ontario, Canada"* published in *Scientific Report* (DOI: 10.1038/s41598-018-24764-7).

1 Overview

We used an *in-silico* population to simulate fairly realistically the spread of pandemic influenza in the province of Ontario, Canada, and evaluate the potential benefits of early vaccine availability. To this end, we developed an agent-based model in order to capture the heterogeneities of transmission dynamics.

The implementation of the agent-based model is *not* event driven. Instead, the time unit (*i.e.*, day) is divided into relatively coarse "slices" that represent specific time-periods during the course of a day for relevant social activities. Within a given time slice, epidemiological events may occur and are computationally accounted for only *once*. This approximation allows significant computing time savings for an acceptable loss of realism (which depends on the size of time slices). Although each individual is uniquely identified, the information that who acquires infection from whom is omitted for performance (however, the time of transmission is always recorded to keep track of the generation interval).

The model is implemented with a coarse spatial structure. The fundamental spatial unit is called a "social place", which broadly represents any physical place that individuals may interact. Because it is preferable to have a limited number of types for social places, only the most relevant ones for which data exist are explicitly represented (*e.g.*, households, schools, workplaces, public transportation). We also used a generic social place type that represents all other types of social places. Social places are not geolocated.

The natural history of influenza and transmission processes for each individual are determined probabilistically. Individuals are assigned age-dependent levels of immunity and frailty. These levels determine the likelihood of disease transmission, symptomatic infection, hospitalization and disease-induced death, as detailed in the following sections.

The programming language for the computational model is C^{++}. The executable is wrapped as a R[26] library for output analysis. The execution time of this computer program, for a single simulation with 50,000 individuals, is about 30 seconds on a single 2.1 GHz AMD Opteron processor.

2 Population

2.1 Social place

The model is implemented with a spatial structure, and its fundamental spatial unit is called a "social place". It represents real world locations where individuals may have relevant contacts for disease transmission (*e.g.*, households, schools, workplace, public transportation). Because

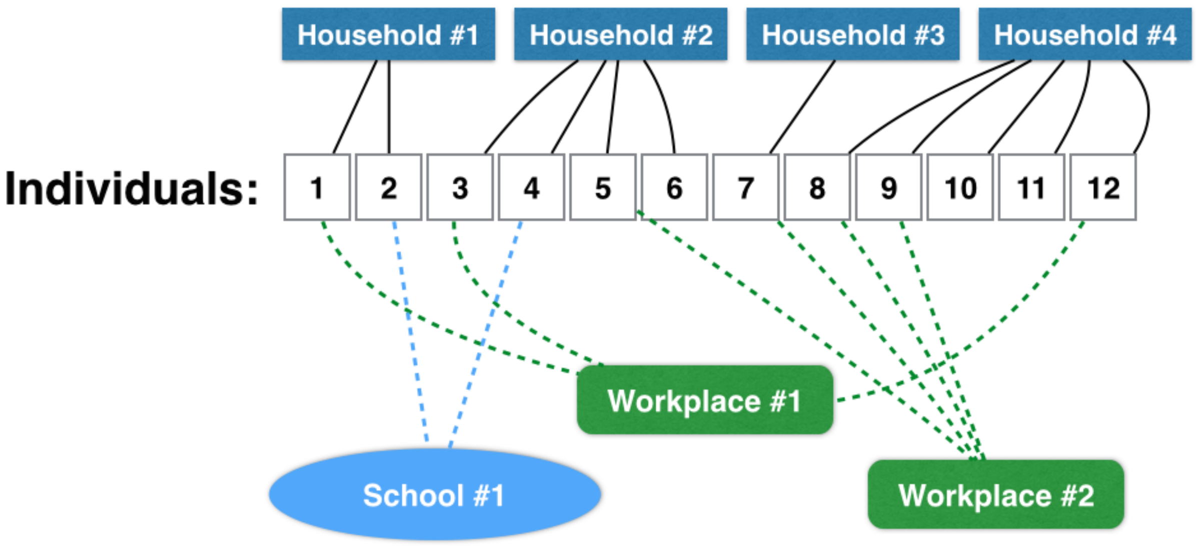

Figure 1. *Schematics diagram for linkage of individuals to social places. All individuals are linked to social places of type "household" (solid link). Links to other social places depends on the individual's characteristics, like age. For example, individual 1 is an employed adult and hence is linked to a workplace, but not a school.*

all types of such social places cannot be explicitly described in the model, a social place typed "other" is used for all other real-world locations. In order to have a similar hierarchical structure as real world data, social places are gathered in "area units", which constitute "regions".

The size of a social place, that is the number of linked individuals, is pre-specified with a distribution given as an input taken from provincial or national statistics, when available (see details in the following sections).

2.2 Individuals

Individuals are uniquely identified in the model with a unique identification number. The modelled characteristics of an individual include infectious status, age, level of immunity against the disease, and frailty. Immunity and frailty are modelled as real numbers between 0 and 1. They determine respectively the risk of acquiring the infection and being hospitalized as a result of infection.

Individuals are "linked" to social places. A link to a social place means that the individuals visit and have contacts in it (according to their schedule, see details later). All individuals are linked to a social place of type "household". Link to other social places depends on the characteristics of individuals: for example, all children between 5 and 18 years of age are linked to a school but none is linked to a workplace (Figure 1).

2.3 *in-silico* Population construction

The population where the simulations will take place is defined iteratively. First, a large number of individuals are created and assigned to households according to the pre-specified distribution

of household sizes. All households have at least one individual. Once all the households are populated, individuals are assigned an age based on pre-specified age distributions conditional on the household size. For example, a household of size one is associated with an age distribution that has a minimum value of 18 years old in order to avoid households populated with only one child. There is no individual without a link to a household.

For this study, the distributions of household sizes and population age profiles in Ontario were retrieved from Statistics Canada (2011 Census, catalogue 98-313-XCB2011022 for the household size and catalogue 98-311-XCB2011023 for ages). We did not have a direct access to the distribution of age of individuals conditional on their household size. However, we obtained the age distribution of the entire population, as well as the distribution of household sizes. Hence, it is possible to reconstruct (albeit, not in a unique fashion) the distributions of age of individuals conditional on their household size.

Let $\phi_{i,j}$ be the distribution of age for the ith oldest individual in a household of size j. We chose to parametrize $\phi_{i,j}$ as a Beta distribution because it allows to restrict ages between a lower- and upper-limit. This is useful, for example, when setting the age of individuals in households of size one, where the lower limit is 18 years and the upper limit is 99 years (hence no household of size one is populated with only a child). The parameters of Beta distributions are fitted such that, combined with the distribution of household sizes, they give the closest age distribution for the whole population (*not* conditional on household size) to the data. The fit is performed using an optimization procedure (function `nlopt` in R).

Construction of other social places is rather straightforward because there is no complex constraints on the age distribution of individuals linked to them. For example, individuals younger then 18 years of age are linked to schools (see section 3.2 for a detailed explanation) . The total number of a given social place and its size distribution are pre-specified as inputs. Then, individuals are assigned randomly to social places based on their schedule. Only social places tagged as "other" do not have individuals linked to them, because their purpose is to be visited randomly.

To summarize, the high level algorithm for the construction of the simulated population includes the following steps:

1. Create N_h empty households.
2. Create N_i individuals without any specific age (with $N_i \gg N_h$).
3. Link households with these individuals according to the distribution of household sizes.
4. Delete superfluous (not linked to any households) individuals.
5. Assign ages to individuals that are linked to a household, based on a pre-specified age distribution given a household size.
6. Create social places according to pre-specified number and size distribution.
7. Link individuals to social places according to their schedules.

The distribution of the sizes of the social places implemented are shown in Figure 2. They

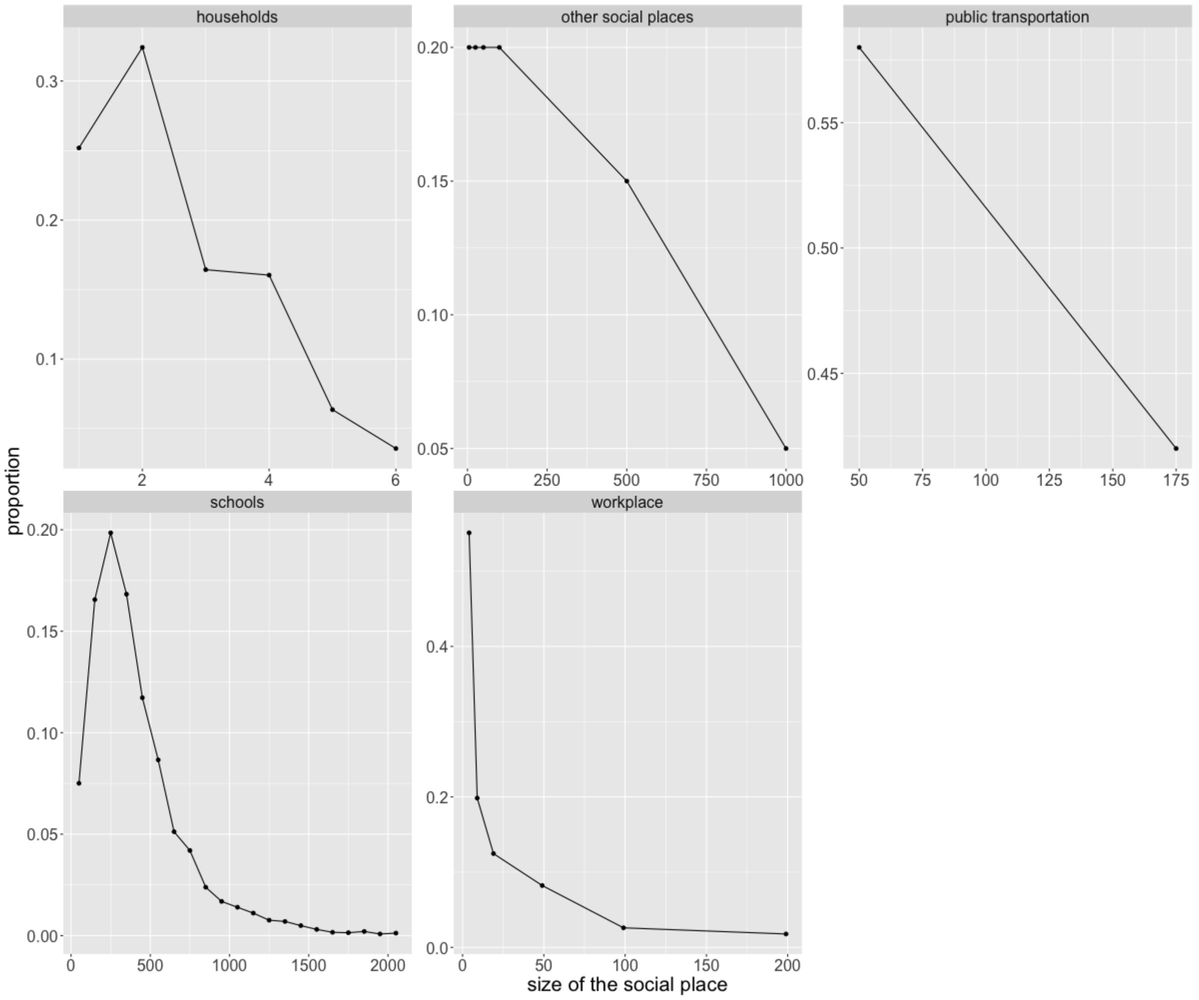

Figure 2. *Distribution of the sizes of the social places used in the simulations. The data were extracted from different databases relevant to Ontario (see text).*

were informed from Statistics Canada for the households and workplaces, from the Toronto Transit Commission for the public transportation, from the Ontario Ministry of Education for the schools, and assumed to arbitrary values for the social places of type "other".

3 Simulator

3.1 Model structure

The model implements, at the individual level, the natural history of influenza. Upon infection, a susceptible individual becomes exposed (E), i.e., infected but not yet infectious. After a period of time (drawn from the distribution of the latent period for each individual), exposed individuals become infectious and will either be symptomatic (I_s) or asymptomatic (I_a). Asymptomatic cases recover (R_a) without any disease outcomes such as hospitalization or death. Symptomatic cases are either recovered (R_s) or hospitalized (H). Hospitalization may lead to either recovery or death (D). Only symptomatic cases are eligible to receive antiviral treatment. All other epidemiological states that do not show symptoms (that is S, E, I_a and R_a) are eligible to receive

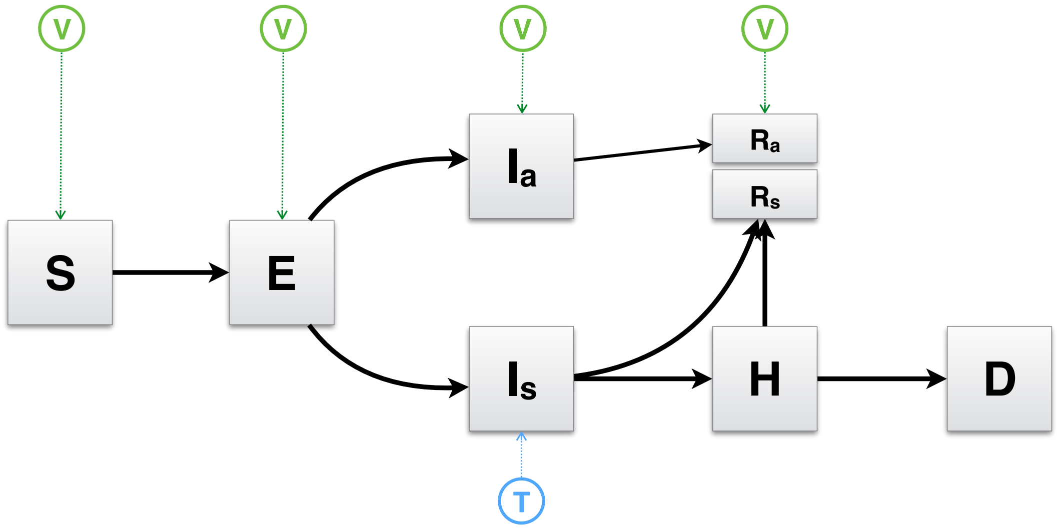

Figure 3. *Compartmental representation of the epidemic model. Upon infection, individuals move from a susceptible (S) state to the exposed state (E) in which they are not infectious yet. Their infection is either symptomatic (I_s) or asymptomatic (I_a). Asymptomatic individuals will recover (R_a) after their infectious period. Symptomatic cases will either recover (R_s) or be hospitalized (H). Hospitalized patients will either recover or die from infection (D). Asymptomatic cases are not hospitalized. Depending on the intervention and its targeted population, vaccine (V) and treatment (T) may be administered at different stages of infection. The type of treatment for hospitalized cases is likely different (and much more intensive) than for non-hospitalized ones. So we considered that the treatment received in a hospital should not be added up with the antiviral one, which explains the absence of a treatment arrow in this diagram.*

influenza vaccine, in addition to any other constraints from the chosen vaccination scenario. Finally, we do not include demographic variables of birth and natural death given the short duration of an influenza pandemic wave. Figure 3 illustrates the disease states.

3.2 Schedules and spatial movements

Every individual is assigned a daily "schedule" that determines which social places are visited at any given time of the day. There is a pre-specified probability that the visit actually occurs (based on the individual characteristics and epidemiological status, symptomatic infection may reduce this probability). For example, there is limited number of contacts if an individual's schedule allows mostly for movements between the assigned household and workplace. But social places like "other" or "public transportation" bring some additional mixing. An illustration of schedules is given in Figure 4.

We defined four types of schedules: "student", "unemployed", "worker" and "worker using public transportation". The schedule type is assigned based on the age of the individual. Individuals younger than 18 had a "student" schedule, those older than 65 had an "unemployed" one. Those of working age (between 18 and 65) are assigned either a "worker" or "unemployed" schedule. The later is chosen with a 0.10 probability. The proportion of workers using public transportation is set at 0.12 (of all the worker population) based on the num-

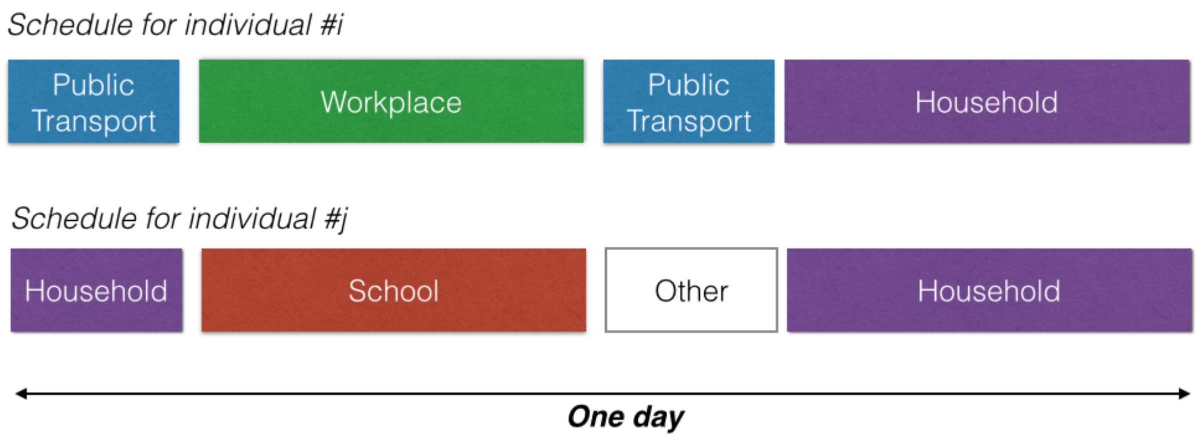

Figure 4. *Simple illustration of different schedules for two individuals. In this example, a day is divided into four unequal time slices. All individuals have the same time partition, but the social place associated to the time slices can be different. In this illustration, individual j visits household and school for the first two time slices of the day. Then visits another social place, randomly chosen, and ends the day by returning to the assigned household.*

bers published by Statistics Canada (http://www12.statcan.gc.ca/nhs-enm/2011/as-sa/99-012-x/2011003/tbl/tbl1a-eng.cfm).

Individuals with symptomatic infection may self-isolate, with a probability set at 0.9 [31], by staying in their household during their infectious period.

3.3 Frailty

Individuals have different levels of vulnerability to influenza infection which influences the risk of infection, severe outcomes, or even death. This vulnerability is represented in the model by a "frailty index" with a value between 0 and 1 (1 corresponds to the highest vulnerability) and depends on age. Individuals at higher risk of influenza-induced complications than the general population include those with chronic respiratory illness, cardiovascular diseases, immunosuppression, and diabetes [22]. Hence, in order to represent the correct age profile, the shape of the frailty index is fitted to survey data of chronic diseases (2014 Canadian Community Health Survey). Once the shape is fitted, the absolute level of the frailty index is fitted to the population hospitalization rate, which is specified in scenarios. We performed a segmented linear regression to fit the frailty shape $f(a)$, as a function of age a (using the `segmented` package in R). Figure 5 shows the fit for frailty index.

For each individual in the simulation, the value of frailty index is drawn from a normal distribution with the mean $f(a)$ (where a is the individual's age) and standard deviation arbitrarily set at 0.1. This deviation was chosen as the maximum difference of frailty between consecutive 5-year age groups provided in data.

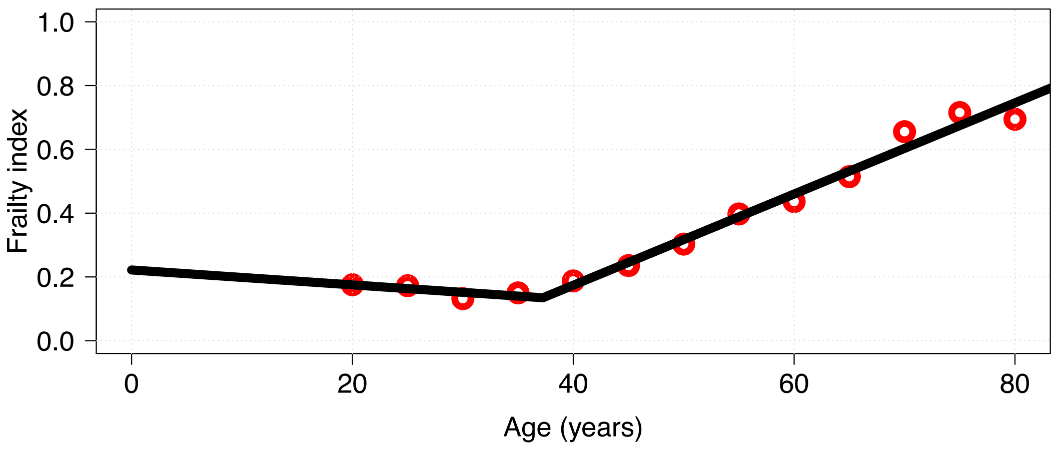

Figure 5. *Frailty index (back line) as a function of individual's age. Red circles show data from the Canadian Community Health Survey 2014, representing the proportion of individuals having a chronic disease.*

3.4 Humoral immunity

Humoral immunity reflects the pre-existing cross-reactive antibodies against the pathogen. The level of this immunity can determine whether or not an infection can successfully establish itself in the host. The level of humoral immunity depends on pre-existing cross-reactive antibodies as a result of prior exposures or vaccination. We label this level $\mathcal{I}_h$ and model it as a real number ranging from 0 to 1, with 1 corresponding to full protection against influenza infection.

In the context of pandemic influenza, individuals will have little to no pre-existing cross-reactive antibodies, so the baseline protective effect of humoral immunity $\mathcal{I}_h$ is assumed to have a negligible value in our model, and we set $\mathcal{I}_h = 0$. However, the protective effect of humoral immunity increases following vaccination. Vaccination leads to the generation of humoral immunity which reaches its maximum level over a 2-week period. This level is determined for each individual based on the frailty index and vaccine efficacy, as detailed in Section 4.3.

3.5 Cellular immunity

Cellular immunity is modelled as a determinant of symptomatic infection, and assumed to be age-dependent. Because this immunity "builds up" through life [13, 12], older individuals are expected to have a higher level of cellular immunity than younger individuals. Thus, the protective effect of cellular immunity for an individual of age a, $\mathcal{I}_c(a)$, is modelled with a logistic shape curve (Figure 6):

$$\mathcal{I}_c(a) = \frac{\mathcal{I}_c^*}{1 + e^{-s(a/a_{pivot}-1)}} \tag{1}$$

where $\mathcal{I}_c^*$ is the maximum level of cellular immunity, and s and a_{pivot} are shape parameters representing the slope and inflection point, respectively. Note that the values for $\mathcal{I}_c$ are bounded by 0 and 1. The parameters s and a_{pivot} are chosen such that the level $\mathcal{I}_c$ is close to the immune

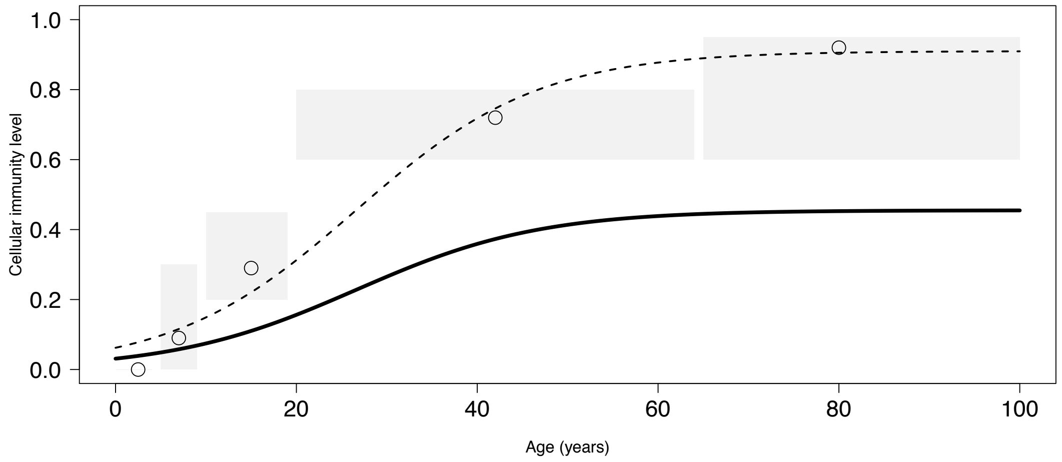

Figure 6. *Dashed line: level representing the protective effect of cellular immunity calibrated on [30]; fitted values are* $\mathcal{I}_c^* = 0.91$, $a_{pivot} = 26.59$ *years and* $s = 2.61$. *Circles (shaded rectangles): estimated median (ranges) from [30]. Solid line: cellular immunity* $\mathcal{I}_c$ *used in the model, obtained by only changing* $\mathcal{I}_c^* = 0.455 = 0.91/2$.

fraction estimated in a serological study of 2009 H1N1 ([30], figure 4). Then, because our baseline pandemic scenario assumes less pre-existing cellular immunity than the 2009 H1N1 pandemic, we arbitrarily divide $\mathcal{I}_c^*$ by 2. The other parameters s and a_{pivot} remain unchanged to keep the shape of the calibrated age-dependent cellular immunity $\mathcal{I}_c$.

3.6 Transmission process

Individuals visit a social place during a period that is determined by the time slots of their schedules. During each visit, interactions with other individuals are modelled only if it involves a potential transmission. The level of protection conferred by humoral and cellular immunity affects disease transmission (Figure 8, equation 4) and the course of disease (equation 5).

3.6.1 Number of contacts

When an infectious individual is present in a social place, a pre-specified contact rate r_c determines the number of contacts n_c with other co-located individuals during the sojourn in that social place. For an individual of age a in a social place s, the number of contacts is drawn from a Poisson distribution with the mean drawn from a lognormal distribution.

$$r_c \sim \log \mathrm{N}(\lambda(a,s),\sigma) \quad (2)$$
$$n_c \sim \mathrm{Poisson}(r_c) \quad (3)$$

The rate r_c is log-normally distributed to allow for super-spreading events[20]. The mean λ of the log-normal distribution is parameterized based on the characteristics of individuals and the social places: it is modelled as the product of a baseline, constant, contact rate Λ and an

adjustment factor $\beta(a,s)$. Parameter Λ is fitted to obtain a specific basic reproduction number $\mathcal{R}_0$ for a given scenario. Values of $\mathcal{R}_0$ (and hence Λ) based on estimates from past epidemics and pandemics [1] are explored in scenarios to reflect different disease transmissibility and severity (see main text of article for scenario descriptions).

Unfortunately, there is no good agreement between empirical studies that investigated the contact rates among different age groups.

Face-to-face contact studies conducted in France [29, 11, 10, 2] found that young children have a much higher contact rate than the rest of the population. Whereas a Canadian study [28] on the timing of laboratory-confirmed influenza infections across several seasons found that teenagers and young adults were infected earlier than other age groups (in particular young children), suggesting the age group 13-24 years old has a larger contact rate. Hence, we decided to fit the age component of $\beta(a,.)$ to Canadian study for our baseline scenario and as a sensitivity analysis, fit it to the French face-to-face contact study. We did not find any study that could reliably inform the relative contact rates between social places, so we set $\beta(.,s) = 1$ for all social places s.

The standard deviation σ is calculated such that the coefficient of variation is constant, arbitrarily fixed at 0.75 (different values are explored in the sensitivity analysis).

3.6.2 Contacts selection

Once the number of contacts from an infectious individual is determined, susceptible individuals in the same social place are selected randomly as contacts, with a constraint on age assortativity.

A predefined function $\varphi(x,y)$ determines the probability that an individual aged x will contact another individual aged y. In a given social place and for a given number of contacts, all susceptible individuals are assigned a "score" to be contacted based on the assortativity function φ. The higher the score, the more likely the individual will be contacted. The selection is stochastic and a parameter ξ enables some deviation from the desired assortativity φ. The scores are sorted and the susceptible contact is selected by choosing the rank of the sorted scores from an exponential distribution. If $S_1, S_2, \ldots$ are the candidate susceptible individuals and u is the indices vector of the ranked scores, the selected susceptible will be $S_{u[X]}$ with $X \sim \text{Exp}(\xi)$. The smaller ξ, the more likely that the choice of the susceptible individual will comply to the predefined assortativity function φ. If the selection of the ranked candidate contacts is deterministic ($\xi = 0$), then age assortativity is too rigid, leading to unrealistic contact patterns.

The function φ was based on the POLYMOD study by Mossong et al [24]. This study was conducted in European countries and no equivalent study is available for Canada. Here, we implicitly assume that the age assortativity in contact patterns in Ontario is similar to that studied in European countries. The pre-specified function φ is shown in Figure 7.

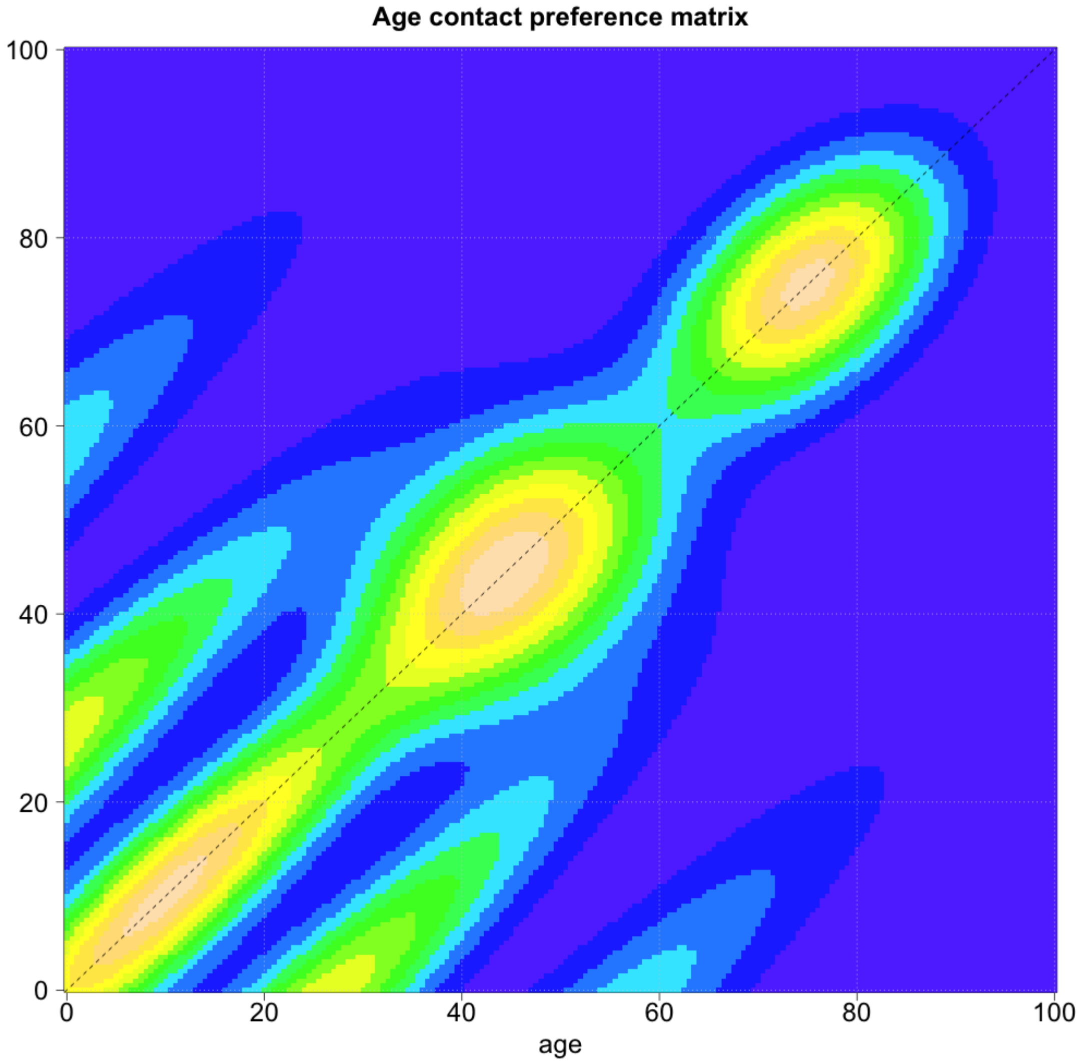

Figure 7. *Age assortativity function φ. Brighter colour indicates a larger probability of contact.*

3.6.3 Transmission probabilities

The probability that a given contact transmits the disease is reduced by a factor of ρ_{asympt} if the infector is asymptomatic. We set $\rho_{\text{asympt}} = 0.1$[18, 27] and explored other levels in the sensitivity analysis.

The probability that a susceptible contact acquires infection is affected by the level of humoral immunity ($\mathcal{I}_h$), which in our model is simply interpreted as the probability that infection fails to establish in the host. The probability to acquire infection, conditional upon an infectious contact, is therefore reduced by a factor

$$p_{\text{acq}} = 1 - \mathcal{I}_h \tag{4}$$

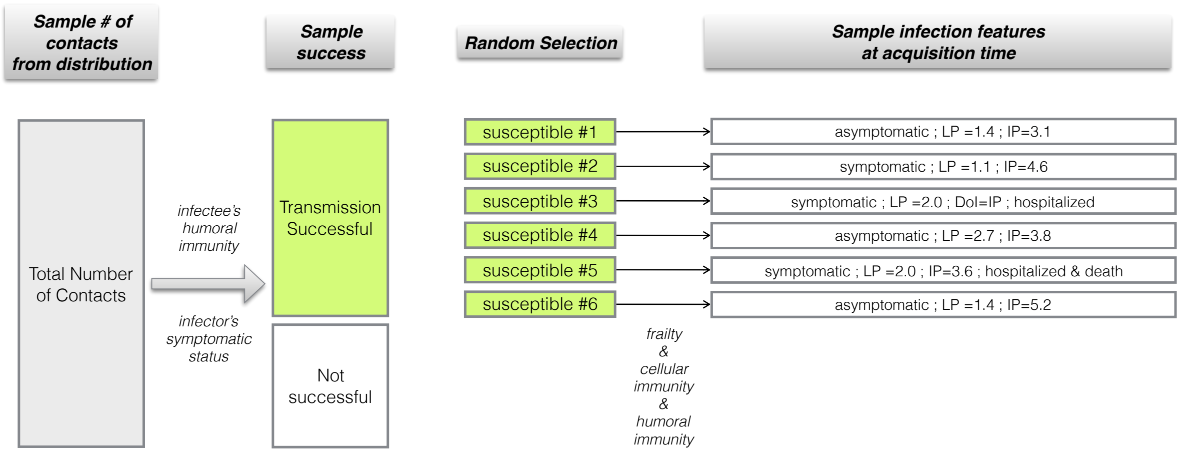

Figure 8. *An illustration of the transmission process for an infectious individual in a given social place. LP: latent period. IP: infectious period.*

3.7 Disease progression

Once an individual has acquired the infection, latent and infectious periods are drawn from lognormal probability distributions with means (variances) of 1.25 (0.25) and 1.5 (1.0) days, respectively [8, 18, 4].

3.7.1 Asymptomatic and symptomatic infections

The symptomatic nature of the infection is determined probabilistically at the time of disease transmission. In the absence of vaccine, the probability of developing symptomatic infection is based on both the frailty index f and the level of cellular immunity $\mathcal{I}_c$:

$$p_{\text{sympt}} = f(1 - \mathcal{I}_c)\mu \tag{5}$$

where μ is a parameter fitted to the asymptomatic fraction (*i.e.*., the proportion of asymptomatic infections among all infections) defined for a given scenario. Note that both the frailty and level of cellular immunity are age dependent.

Vaccination can also reduce the probability of developing symptomatic infection [3, 6]. See section 4.3 for a description of the implementation of this mechanism.

3.7.2 Hospitalization and death

If an individual develops symptomatic infection, then there is a risk of hospitalization and subsequently, a risk of death. For any symptomatic infection, the hospitalization event is drawn from a Bernoulli distribution with the probability p_{hosp} that depends on the individual's frailty f as follows:

$$p_{hosp}(f) = f\alpha_h \tag{6}$$

Parameter α_h affects the overall number of influenza-induced hospitalizations and the frailty f affects the age distribution of hospitalizations.

If hospitalization occurs, then the duration of the infectious period before hospitalization (i.e., the time to hospitalization) is sampled from a discrete distribution from Table 3 of Morrison *et al.* [23]. The duration of hospitalization is drawn from a lognormal distribution with the mean d_{hosp} set at 12 days and variance 21 days^2 [16].

Death event conditional upon hospitalization is drawn from a Bernoulli trial with a probability that depends on frailty:

$$p_{death}(f) = f\alpha_d \tag{7}$$

Parameter α_d affects the overall number of influenza-induced deaths and the frailty f affects the age distribution of mortality.

The parameters α_h and α_d are obtained by fitting to the hospitalization and death probabilities specified in scenario assumptions. If $\bar{p}_h$ is the pre-specified hospitalization probability for a given scenario, $f(a)$ is the average frailty index for individuals aged a, and $\pi(a)$ is the proportion of individuals with age a in the whole population, the fitted value for α_h is given by

$$\hat{\alpha}_h = \frac{\bar{p}_h}{\sum_a f(a)\pi(a)} \tag{8}$$

Similarly, for the probability of death conditional upon hospitalization, we calculate

$$\hat{\alpha}_d = \frac{\bar{p}_d}{\sum_a f(a)\pi(a)} \tag{9}$$

The hospitalization and mortality probabilities chosen in our scenarios were based on the corresponding data obtained from Public Health Agency of Canada [25]

Hospitalized individuals are moved to a linked social place labelled as “hospital” and stay in hospital for the duration of hospitalization (i.e., movements to other social places directed by their schedule are ignored during hospitalization).

Figure 9 provides a diagram for the progression through various disease states.

4 Interventions

4.1 Deployment

Antiviral treatment and vaccination are implemented as part of “interventions”. There can be several simultaneous interventions of different nature (e.g., vaccination, antiviral treatment). All interventions are modelled as having the following features:

- **type:** defines the nature of the intervention: treatment, vaccination, behavioural change (self-isolation).
- **target population:** defines the group of individuals that may receive the intervention.

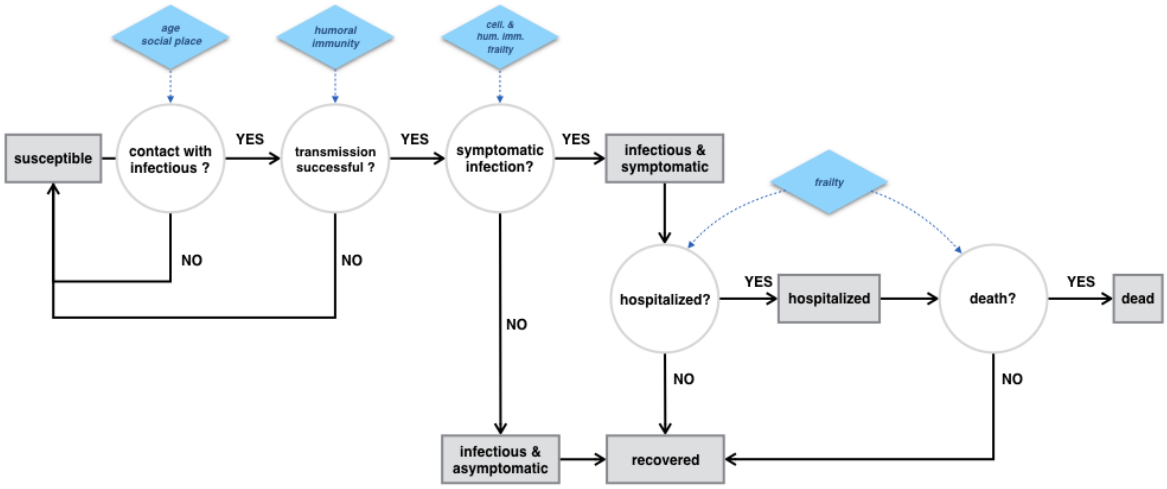

Figure 9. *Diagram for the progression through disease states. Shaded rectangles are disease states, white circles represent transitions between disease states. Blue lozenges indicate the individuals' characteristics that affect the progression of the disease.*

- **time range:** when the intervention begins and ends.
- **administration rate:** defines the fraction of population that receives the intervention during a given period of time.
- **maximum coverage:** specifies if there is a limit to the total number of individuals receiving the intervention.

Although the target groups in the population and administration rates are deterministic, the actual number of individuals receiving an intervention is stochastic. If T is the total number of individuals targeted to receive the intervention, r is the coverage, and dt is the simulation time step, then the actual number N_{dt} of individuals receiving the intervention during the time step dt is

$$N_{dt} \sim \text{Poisson}(r\,T\,dt) \tag{10}$$

Moreover, the total number of individuals receiving the intervention is monitored at every time step of the simulation such that the constraint specified by the maximum coverage is fulfilled.

4.2 Antiviral treatment

Literature on the effects of antiviral treatment (*i.e.*, neuraminidase inhibitors) during the course of influenza infection indicates that it marginally reduces the duration of symptoms. It is unclear if antiviral treatment reduces the risk of severe complications, hospitalization or even death [15, 14].

Hence, our model implements the effect of antiviral treatment for symptomatic infection as reducing the infectious period. We considered a period of 12 hours as the minimum delay in start of the treatment following the onset of symptoms. This delay may reflect the time lag to

seek care and receive antiviral treatment. Furthermore, we assume that individuals (who are not hospitalized) will not receive antiviral treatment if the time since the onset of symptoms has elapsed 2.5 days. This reflect the fact that antiviral treatment is most effective if initiated within 2 days after the onset of symptoms. More specifically, if d is the infectious period drawn from the pre-specified distribution at the time of disease transmission, the reduction of infectious period (δ) due to antiviral treatment is modelled as a function of the time since symptom onset τ (in days):

$$\delta(\tau) = (2.5 - \tau)/2, \qquad 0.5 < \tau < 2.5 \tag{11}$$

This formulation indicates that if antiviral treatment starts at its earliest time (i.e., half a day) after the onset of symptoms, the infectious period reduces by a maximum of 1 day [15]. This reduction decreases with further delay in start of the treatment. In our model, antiviral drugs are considered for the treatment of symptomatic cases only, and not prophylaxis.

4.3 Vaccination

4.3.1 Effects of vaccine on disease transmission

Vaccination is modelled as potentially increasing the protective effect of humoral immunity and decreasing the risk of developing symptomatic infection [5].

Following vaccination, each individual will have its humoral immunity level $\mathcal{I}_h$ increased to 1.0 (its maximum value) with a probability p_{eff} that represents the vaccine efficacy at the individual level. We model this efficacy as dependent on frailty:

$$p_{\text{eff}}(f) = (1 - f)\varepsilon \tag{12}$$

where parameter ε is the maximum vaccine efficacy for an individual that is fully immune-competent. Values for ε are set on a scenario basis within the estimated ranges in the published studies [7].

The increase in the level of humoral immunity following vaccination is not instantaneous, but increases linearly to its maximum level (*i.e.* 1.0) during a 2-week period [5]. We assumed this maximum level of protective immunity is reached with one dose of vaccine only. Influenza vaccination is assumed to have no effect on individuals' frailty.

Vaccination is shown to reduce the risk of developing a symptomatic infection and severe outcomes, even if it fails to fully protect against infection [3, 6]. We model this reduction based on the vaccine efficacy at the individual level [17]. Thus, the probability of symptomatic infection p_{sympt} for a vaccinated individuals (defined in equation (5)) is multiplied by a factor $1 - p_{\text{eff}}(f)$ if the individual is infected after the vaccine-induced immunity has reached its maximum level. See Figure 10 for an illustration of the vaccination process.

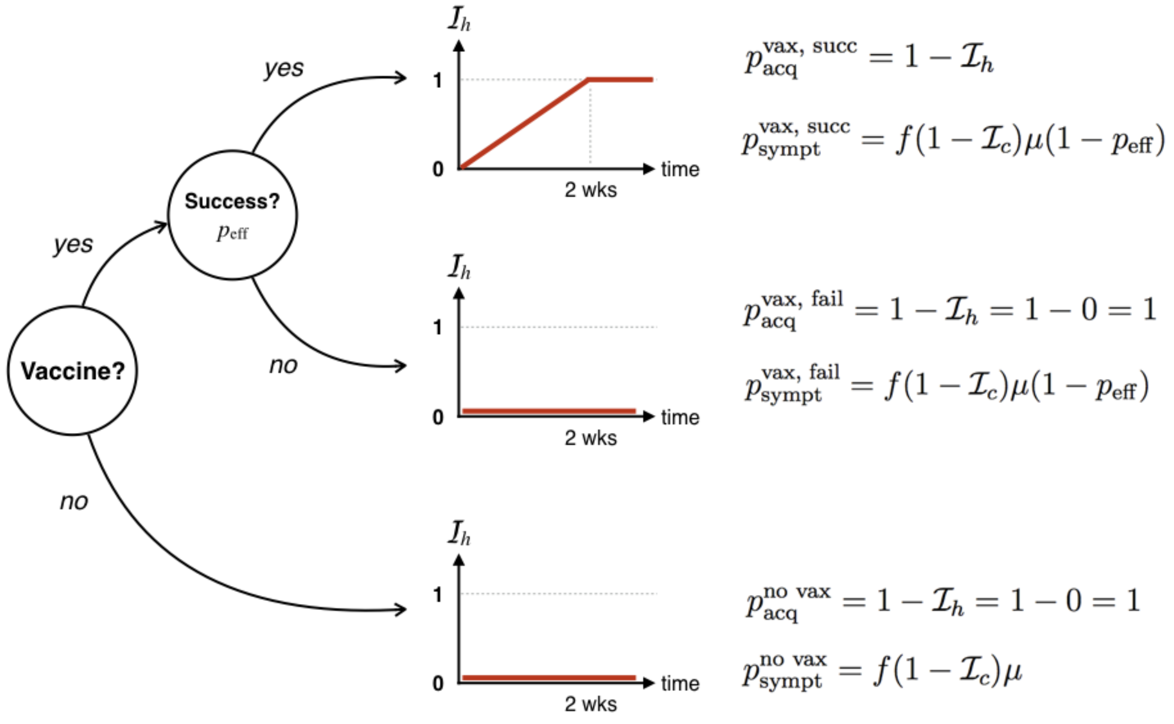

Figure 10. *Illustration of the vaccination process in the model and its effect on the level of humoral immunity $\mathcal{I}_h$, the probability of influenza acquisition p_{acq} and the probability of symptomatic infection p_{sympt}.* ***Top panel:*** *a successful vaccination occurs with probability p_{eff} and increases, linearly over 2 weeks, the level of humoral immunity $\mathcal{I}_h$ to 1.0. Moreover, the risk of developing a symptomatic infection is reduced.* ***Middle panel:*** *if the vaccine fails to provide immunity, the level of immunity $\mathcal{I}_h$ remains at 0 and the individual is still susceptible to infection. However, the vaccine still reduces the risk of developing a symptomatic infection.* ***Bottom panel:*** *an unvaccinated individual is fully susceptible to infection and its probability to develop symptom upon infection is unchanged.*

4.3.2 Vaccination strategy

We implemented a vaccination strategy inspired from the influenza pandemic planning strategy in Ontario (Ontario Health Plan for an Influenza Pandemic, 2013, chapter 7, immunization). This planning strategy assumes the vaccine is not available during the first months of the pandemic, whereas this study explores early (even before the first imported case) availability. The vaccination strategy implemented in the model prioritizes specific age groups and individuals in high-risk groups (*i.e.*, patients with pre-existing chronic conditions as identified by the National Advisory Committee on Immunization). More specifically, the vaccination strategy is driven by the following algorithm:

1. The priority population group consists of individuals:
   - aged 5 years and younger
   - aged 65 years and older
   - with a frailty index f larger than the population average frailty $\bar{f}$
2. As long as the average vaccination proportion within the priority population group is below

35%, continue with prioritizing these groups

3. Once the average vaccination proportion within the priority population groups reaches 35%, other individuals can also be vaccinated.

4. The vaccination proportion is capped by age (even for the priority group) at pre-specified levels.The maximum proportion of vaccinated individuals by age were based on a study conducted in Ontario following the 2009 H1N1 pandemic influenza [9]: 45% for ages 12 and younger, 25% for ages between 12 and 30 years, 35% for ages between 30 and 55 years, and 55% for ages 55 years and older.

4.3.3 Vaccine administration rate

The rate of vaccination (in terms of vaccines administered per day) for seasonal influenza can be approximated by knowing the proportion of a population that is vaccinated and the vaccination roll-out period. For example, assuming that 30% of the population is vaccinated during a 3-month period, the average vaccination rate is 300 per 100,000 people per day (30%/90 days). This approximation can be a starting point for the vaccination rate of a pandemic influenza. We investigated fractions and multiples of this level to reflect the shortage of vaccine supply or enhanced public health efforts in vaccine distribution.

5 Table of main model parameters

Table 1. *Main parameters of the agent-based model.*

Category	Name in C^{++}code	Symbol	Description	Value	Unit	Sources
Nat. Hist.	`dol_distrib`	-	Distribution latency duration	lognorm		Arbitrary
Nat. Hist.	`dol_mean`	-	Duration of latency, mean	1.25	day	[8, 18, 4]
Nat. Hist.	`dol_var`	-	Duration of latency, variance	0.25	day^2	[8, 18]
Nat. Hist.	`doi_distrib`	-	Distribution infectiousness duration	lognorm		Arbitrary
Nat. Hist.	`doi_mean`	-	Duration of infectiousness mean	3.0	day	[8, 18, 4]
Nat. Hist.	`doi_var`	-	Duration of infectiousness variance	1	day^2	[8, 18]
Nat. Hist.	`proba_hosp`	α_h	Hospitalization proba multiplier	fitted		PHO
Nat. Hist.	`proba_death_mult`	α_d	Death proba parameter	fitted		PHO
Nat. Hist.	`doh_distrib`	-	Distribution of duration of hospitalization	lognorm		[16]
Nat. Hist.	`doh_mean`	-	Duration of hospitalization, mean	12	day	[16],table 4
Nat. Hist.	`doh_var`	-	Duration of hospitalization, variance	21	day^2	[16]
Nat. Hist.	`mult_proba_symptomatic`	μ	Determine (indirectly) overall asymptomatic fraction	fitted		[19]
Nat. Hist.	`asymptom_infectiousness_ratio`	ρ_{asympt}	Reduction of infectiousness when asymptomatic (vs symptomatic)	0.1		[18, 21]
Biology	`frailty_0`	-	Frailty index for infants (age $\leq$ 1 year-old)	fitted		PHAC [25]
Biology	`frailty_agepivot`	-	Age when the segmented regression changes slope	fitted	year	PHAC [25]
Biology	`frailty_slope1`	-	Slope of segmented regression for ages < `frailty_agepivot`	fitted		PHAC [25]
Biology	`frailty_slope2`	-	Slope of segmented regression for ages > `frailty_agepivot`	fitted		PHAC [25]
Biology	`frailty_sd`	-	Frailty index standard deviation when allocated to individual	0.1		PHAC [25]
Biology	`imm_hum_baseline`	$\mathcal{I}_h$	Baseline value for humoral immunology	0.0		scenario assumption. May be increased following vaccination.
Biology	`imm_cell_max`	$\mathcal{I}_c^*$	Cellular immunity max value	fitted		0.91 based on [30]
Biology	`imm_cell_slope`	s	Cellular immunity shape parameters	fitted		2.61 based on[30]
Biology	`imm_cell_pivot`	a_{pivot}	Cellular immunity shape parameters	fitted	year	26.59 based on[30]
Behaviour	`contact_rate_mean`	Λ	Mean of contact rate distribution	fitted	contacts per capita per day	scenario assumption on the basic reproduction number $\mathcal{R}_0$,[1]
Behaviour	`contact_rate_stddev`	σ	Standard deviation of contact rate distribution	fitted		Value chosen such that the coefficient of variation is 0.75
Behaviour	`contact_ratio_age_1_10`	$\beta(i,s)$	Ratio for contact rate for indiv aged 1 to 10 yrs old	10		[29, 11]
Behaviour	`contact_ratio_age_10_16`	$\beta(i,s)$	Ratio for contact rate for indiv aged 10 to 16 yrs old	3		[10, 2]
Behaviour	`contact_ratio_age_over_65`	$\beta(i,s)$	Ratio for contact rate for indiv aged > 65	1		Arbitrary
Behaviour	`contactAssort_lambda`	ϕ	Contact assortativity parameter (the larger, the more associative according to matrix input)	0.1		Arbitrary
Behaviour	`unemployed_prop`	-	Proportion of unemployed individuals aged between 18 and 65	0.1		Statistics Canada
Behaviour	`pubT_prop`	-	Proportion of working individuals using public transports	0.12		Toronto Transit Commission
Mouvement	`proba_move`	-	Probability to move to the next social place	0.98		Arbitrary
Mouvement	`proba_move_reduc_sympt`	-	Reduction multiplier for probability to move when symptomatic infection	0.1		[31], Table 1
Mouvement	`proba_change_sp_other`	-	Probability to change 'other' social place during a time slice.	0.2		Arbitrary
Intervention	`treat_doi_reduc`	δ	Mean reduction of duration of infectiousness when antiviral treatment	1	day	[15]
Intervention	`treat_reduc_infect_mean`	-	Mean relative reduction of infectiousness when antiviral treatment	0.1		[15]
Intervention	`vax_imm_hum_incr`	$\mathcal{I}_h$	Increase of humoral immunity index after vaccination	1		scenario assumption. Increase depends on vaccine efficacy.
Intervention	`vax_lag_full_efficacy`	-	Time lag to reach new immunity levels after vaccination	14	day	scenario assumption

References

[1] Matthew Biggerstaff, Simon Cauchemez, Carrie Reed, Manoj Gambhir, and Lyn Finelli. Estimates of the reproduction number for seasonal, pandemic, and zoonotic influenza: a systematic review of the literature. *BMC infectious diseases*, 14(1):480, 2014.

[2] L Bioglio and M Génois. Recalibrating disease parameters for increasing realism in modeling epidemics in closed settings. *BMC Infectious ...*, 2016.

[3] J Castilla, P Godoy, A Dominguez, I Martinez-Baz, J Astray, V Martin, M Delgado-Rodriguez, M Baricot, N Soldevila, J M Mayoral, J M Quintana, J C Galan, A Castro, F Gonzalez-Candelas, O Garin, M Saez, S Tamames, T Pumarola, for the CIBERESP Cases and Controls in Influenza Working Group Spain, E Azor, J Carrillo, R Moyano, J A Navarro, M Vazquez, F Zafra, M F Bautista, J M Navarro, I Pedrosa, M Perez, V Gallardo, E Perez, J R Maldonado, A Morillo, M C Ubago, D Carriedo, F Diez, I Fernandez, S Fernandez, J Castrodeza, C Rodriguez, P Sanz, R Ortiz de Lejarazu, A Perez, P Redondo, A Seco, A Pueyo, J L Viejo, T Fernandez, A Molina, F Barbe, L Blanch, G Navarro, X Bonfill, J Lopez-Contreras, V Pomar, M T Puig, E Borras, A Martinez, N Torner, F Calafell, J Alonso, J Cayla, C Tortajada, I Garca, J Ruiz, J J Garcia, J Gea, J P Horcajada, N Hayes, F Moraga, J Dorca, A Agusti, A Trilla, A Vilella, R Genova, M Garcia Barquero, E Gil, S Jimenez, F Martin, M L Martinez, S Sanchez, R Canton, A Robustillo, C Alvarez, A Hernandez, F Pozo, J R Pano, A Martinez, L Martinez, M Ruiz, P Fanlo, F Gil, V Martinez-Artola, M E Ursua, M Sota, M T Virto, J Gamboa, F Perez-Afonso, U Aguirre, A Caspelastegui, P P Espana, S Garcia, J Aristegui, A Bilbao, A Escobar, I Astigarraga, J M Antonana, G Cilla, J Korta, E Perez Trallero, J L Lobo, F J Troya, and M Morales. Influenza Vaccine Effectiveness in Preventing Outpatient, Inpatient, and Severe Cases of Laboratory-Confirmed Influenza. *Clinical Infectious Diseases*, 57(2):167–175, June 2013.

[4] A Cori, Alain-Jacques Valleron, F. Carrat, G Scalia Tomba, G Thomas, and P Y Boelle. Estimating influenza latency and infectious period durations using viral excretion data. *Epidemics*, 4(3):132–138, August 2012.

[5] R J Cox, K A Brokstad, and P Ogra. Influenza virus: immunity and vaccination strategies. Comparison of the immune response to inactivated and live, attenuated influenza vaccines. *Scandinavian journal of immunology*, 59(1):1–15, January 2004.

[6] Robert G Deiss, John C Arnold, Wei-Ju Chen, Sara Echols, Mary P Fairchok, Christina Schofield, Patrick J Danaher, Erin McDonough, Michelande Ridoré, Deepika Mor, Timothy H Burgess, and Eugene V Millar. Vaccine-associated reduction in symptom severity among patients with influenza A/H3N2 disease. *Vaccine*, pages 1–8, December 2015.

[7] V Demicheli, T Jefferson, and L A Al-Ansary. Vaccines for preventing influenza in healthy adults. *The Cochrane Collaboration*, (3), 2014.

[8] N M Ferguson, D.A.T. Cummings, S Cauchemez, C. Fraser, S Riley, A. Meeyai, S. Iamsirithaworn, and D.S. Burke. Strategies for containing an emerging influenza pandemic in Southeast Asia. *Nature*, 437(7056):209–214, 2005.

[9] Julie Foisy, Laura C Rosella, Ruth Sanderson, Jemila Seid Hamid, Badal Dhar, and Natasha S Crowcroft. Self-reported pH1N1 influenza vaccination coverage for Ontario. *Health reports*, 22(3):29–33, September 2011.

[10] Julie Fournet and Alain Barrat. Contact Patterns among High School Students. *PLoS ONE*, 9(9):e107878, September 2014.

[11] M Génois, C L Vestergaard, J Fournet, A Panisson, I Bonmarin, and Alain Barrat. Data on face-to-face contacts in an office building suggest a low-cost vaccination strategy based on community linkers. *Network Science*, 3(3):326–347, 2015.

[12] K M Gostic, M Ambrose, and M Worobey. Potent protection against H5N1 and H7N9 influenza via childhood hemagglutinin imprinting. *Science*, 2016.

[13] Jason A Greenbaum, Maya F Kotturi, Yohan Kim, Carla Oseroff, Kerrie Vaughan, Nima Salimi, Randi Vita, Julia Ponomarenko, Richard H Scheuermann, Alessandro Sette, and Bjoern Peters. Pre-existing immunity against swine-origin H1N1 influenza viruses in the general human population. *Proceedings of the National Academy of Sciences*, 106(48):20365–20370, December 2009.

[14] T Jefferson, M Jones, P Doshi, E A Spencer, I Onakpoya, and C J Heneghan. Oseltamivir for influenza in adults and children: systematic review of clinical study reports and summary of regulatory comments. *BMJ*, 348(apr09 2):g2545–g2545, April 2014.

[15] Tom Jefferson, Mark A Jones, Peter Doshi, Chris B Del Mar, Rokuro Hama, Matthew J Thompson, Elizabeth A Spencer, Igho J Onakpoya, Kamal R Mahtani, David Nunan, Jeremy Howick, and Carl J Heneghan. Neuraminidase inhibitors for preventing and treating influenza in adults and children. *Cochrane Database of Systematic Reviews*, 2014.

[16] A Kumar, R Zarychanski, R Pinto, and D J Cook. Critically ill patients with 2009 influenza A (H1N1) infection in Canada. *JAMA*, 2009.

[17] M Laskowski, Y Xiao, N Charland, and S M Moghadas. Strategies for Early Vaccination-During Novel Influenza Outbreaks. *Scientific Reports*, pages 1–13, December 2015.

[18] Lincoln L H Lau, Benjamin J Cowling, Vicky J Fang, Kwok Hung Chan, Eric H Y Lau, Marc Lipsitch, Calvin K Y Cheng, Peter M Houck, Timothy M Uyeki, J S Malik Peiris, and Gabriel M Leung. Viral Shedding and Clinical Illness in Naturally Acquired Influenza Virus Infections. *The Journal of Infectious Diseases*, 201(10):1509–1516, May 2010.

[19] Nancy H L Leung, Cuiling Xu, Dennis K M Ip, and Benjamin J Cowling. The Fraction of Influenza Virus Infections That Are Asymptomatic. A systematic review and meta-analysis. *Epidemiology*, 26(6):862–872, November 2015.

[20] J O Lloyd-Smith, S J Schreiber, P E Kopp, and W M Getz. Superspreading and the effect of individual variation on disease emergence. *Nature*, 438(7066):355–359, November 2005.

[21] Ira M Longini, Jr., M Elizabeth Halloran, Azhar Nizam, and Yang Yang. Containing pandemic influenza with antiviral agents. *American Journal of Epidemiology*, 159(7):623–633, April 2004.

[22] D Mertz, T H Kim, J Johnstone, P P Lam, M Science, S P Kuster, S A Fadel, D Tran, E Fernandez, N Bhatnagar, and M. Loeb. Populations at risk for severe or complicated influenza illness: systematic review and meta-analysis. *BMJ*, 347(aug23 1):f5061–f5061, August 2013.

[23] Kathryn T Morrison, David L Buckeridge, Yanyu Xiao, and Seyed M Moghadas. Health & Place. *Health & Place*, 26(C):53–59, March 2014.

[24] J Mossong, N Hens, M Jit, P Beutels, K Auranen, R Mikolajczyk, M Massari, S Salmaso, G S Tomba, and J Wallinga. Social contacts and mixing patterns relevant to the spread of infectious diseases. *PLoS Medicine*, 5(3):e74, 2008.

[25] Public Health Agency of Canada . FluWatch. *Open Data*, 2017.

[26] R Core Team. *R: A Language and Environment for Statistical Computing.*

[27] Eva Santermans, Kim Van Kerckhove, Amin Azmon, W John Edmunds, Philippe Beutels, Christel Faes, and Niel Hens. Structural differences in mixing behavior informing the role of asymptomatic infection and testing symptom heritability . *Mathematical Biosciences*, 285(C):43–54, March 2017.

[28] D Schanzer, J Vachon, and L Pelletier. Age-specific Differences in Influenza A Epidemic Curves: Do Children Drive the Spread of Influenza Epidemics? *American Journal of Epidemiology*, 174(1):109–117, June 2011.

[29] Juliette Stehlé, Nicolas Voirin, Alain Barrat, Ciro Cattuto, Lorenzo Isella, Jean-François Pinton, Marco Quaggiotto, Wouter Van den Broeck, Corinne Régis, Bruno Lina, and Philippe Vanhems. High-Resolution Measurements of Face-to-Face Contact Patterns in a Primary School. *PLoS ONE*, 6(8):e23176, August 2011.

[30] D E te Beest, P J Birrell, J Wallinga, D De Angelis, and M van Boven. Joint modelling of serological and hospitalization data reveals that high levels of pre-existing immunity and school holidays shaped the influenza A pandemic of 2009 in The Netherlands. *Journal of The Royal Society Interface*, 12(103):20141244–20141244, December 2014.

[31] K Van Kerckhove, N Hens, W J Edmunds, and K T D Eames. The Impact of Illness on Social Networks: Implications for Transmission and Control of Influenza. *American Journal of Epidemiology*, 178(11):1655–1662, November 2013.
